# Comparative and kinetic analysis of viral shedding and immunological responses in MERS patients representing a broad spectrum of disease severity

**DOI:** 10.1038/srep25359

**Published:** 2016-05-05

**Authors:** Chan-Ki Min, Shinhye Cheon, Na-Young Ha, Kyung Mok Sohn, Yuri Kim, Abdimadiyeva Aigerim, Hyun Mu Shin, Ji-Yeob Choi, Kyung-Soo Inn, Jin-Hwan Kim, Jae Young Moon, Myung-Sik Choi, Nam-Hyuk Cho, Yeon-Sook Kim

**Affiliations:** 1 Department of Microbiology and Immunology, Seoul National University College of Medicine, Seoul, Republic of Korea; 2 Department of Biomedical Sciences, Seoul National University College of Medicine, Seoul, Republic of Korea; 3 Division of Infectious Diseases, Department of Internal Medicine, Chungnam National University School of Medicine, Daejeon, Republic of Korea; 4 Cancer Research Institute, Seoul National University College of Medicine, Seoul, Republic of Korea; 5 Department of Pharmaceutical Science, College of Pharmacy, Kyung Hee University, Seoul, Republic of Korea; 6 Department of Radiology, Chungnam National University School of Medicine, Daejeon, Republic of Korea; 7 Division of Pulmonology and Critical Care Medicine, Department of Internal Medicine, Chungnam National University School of Medicine, Daejeon, Republic of Korea; 8 Institute of Endemic Disease, Seoul National University Medical Research Center and Bundang Hospital, Seoul, Republic of Korea

## Abstract

Despite the ongoing spread of MERS, there is limited knowledge of the factors affecting its severity and outcomes. We analyzed clinical data and specimens from fourteen MERS patients treated in a hospital who collectively represent a wide spectrum of disease severity, ranging from mild febrile illness to fatal pneumonia, and classified the patients into four groups based on severity and mortality. Comparative and kinetic analyses revealed that high viral loads, weak antibody responses, and lymphopenia accompanying thrombocytopenia were associated with disease mortality, whereas persistent and gradual increases in lymphocyte responses might be required for effective immunity against MERS-CoV infection. Leukocytosis, primarily due to increased neutrophils and monocytes, was generally observed in more severe and fatal cases. The blood levels of cytokines such as IL-10, IL-15, TGF-β, and EGF were either positively or negatively correlated with disease mortality. Robust induction of various chemokines with differential kinetics was more prominent in patients that recovered from pneumonia than in patients with mild febrile illness or deceased patients. The correlation of the virological and immunological responses with disease severity and mortality, as well as their responses to current antiviral therapy, may have prognostic significance during the early phase of MERS.

The Middle East respiratory syndrome coronavirus (MERS-CoV) is an emerging zoonotic pathogen that causes acute and severe respiratory illness with a high mortality rate^1^. Since 2012, more than 1,600 patients have been reported and the mortality rate approaches 35%^2^. Primary transmission of MERS-CoV might be mediated by close contact between humans and infected animal reservoirs such as camels^3^,^4^. However, in Middle Eastern countries, most MERS cases are associated with human-to-human spread starting in healthcare settings that then spark sporadic outbreaks^5^. An unexpected large outbreak in South Korea (186 confirmed cases with 38 deaths), initiated by an infected traveler from the Arabian peninsula, was also attributed to nosocomial infections^6^ and highlights our limited knowledge of this emerging infectious disease^7^.

The major symptoms of MERS cases are acute viral pneumonia often associated with extrapulmonary manifestations such as enteric illness^5^. Patients infected with MERS-CoV present with a wide range of clinical severity varying from asymptomatic to severe pneumonia with respiratory failure^5^. Mortality mainly results from acute respiratory distress syndrome (ARDS)^4^,^5^,^8^. Currently, the pathogenesis of the pulmonary and extrapulmonary manifestations of MERS remains poorly defined and knowledge of factors affecting disease severity is limited, although underlying illness, older age, and high viral loads are associated with poorer outcomes^5^,^8^,^9^,^10^.

Since the outbreak of MERS in South Korea was initiated by an infected person, the clinical courses and epidemiological features, including exposure periods, are well documented for most cases^6^,^11^. Most patients that developed respiratory illness received a combined antiviral therapy composed of pegylated interferon (IFN)-α, ribavirin, and lopinavir/ritonavir, a treatment with unknown efficacy^12^,^13^. We sought to identify the factors dictating disease severity and the outcomes of patients treated with antiviral regimens. Here, we retrospectively analyzed clinical data from fourteen hospitalized MERS patients who collectively represent a wide spectrum of disease severity, ranging from mild febrile illness to fatal pneumonia. In addition, we investigated virological and immunological features of the patients using clinical samples acquired during various stages of MERS progression. Comparative and kinetic analyses may provide valuable insight into the critical factors affecting disease progression and severity as well as the underlying mechanisms contributing to MERS pathogenesis.

## Results

### Clinical characteristics of MERS-CoV patientss

We reviewed all available clinical and laboratory data of fourteen patients treated in a hospital during the MERS outbreak. The patients were classified into four groups based on the severity and mortality (Table 1, Supplementary Fig. S1, and Supplementary Table S1). Group I patients includes two patients who only developed fever and recovered without developing pneumonia. They recovered without any treatment. Group II includes three patients (P03–P05) who developed mild pneumonia without hypoxemia (Table 1 and Supplementary Table S1). P04 and P05 showed elevated C-reactive protein (CRP, >3 mg/dl) and P05 had elevated levels of aspartate aminotransferase (AST), alanine aminotransferase (ALT), and lactate dehydrogenase (LDH) (Supplementary Table S1). Four patients (P06–P09) recovered from more prolonged and severe pneumonia, and are classified as group III. Severe pneumonia was defined as pneumonia severity index (PSI) ≥ 60 at initial presentation (Table 1). All patients in this group exhibited elevated liver enzymes and proteinuria during the acute phase. Among them, P09 had pneumonia, rapidly progressing to respiratory failure, and required mechanical ventilation (MV) and extracorporeal membrane oxygenation (ECMO). She also received convalescent plasma therapy twice on days 10 and 16 after symptom onset (Supplementary Fig. 1). Group IV includes five fatal cases (P10–P14). All group IV patients suffered from severe pneumonia and ARDS requiring high flow nasal cannula and/or mechanical ventilation. The deceased patients were older than 60, except for P11, and had underlying illnesses, except for P10. P11 had decompensated liver cirrhosis and poorly controlled blood glucose levels before MERS-CoV infection. P12 was diagnosed with active pulmonary tuberculosis about one week before he encountered a MERS-CoV infected patient. He developed pneumonia two days after the onset of fever and progressed to ARDS within a week. P13 and P14 had comorbidities including chronic pulmonary obstructive disease (COPD) and asthma. They were infected with MERS-CoV while being treated for an acute exacerbation of their pulmonary diseases. They died within 2 weeks after symptom onset despite mechanical ventilation.

**Figure 1:**
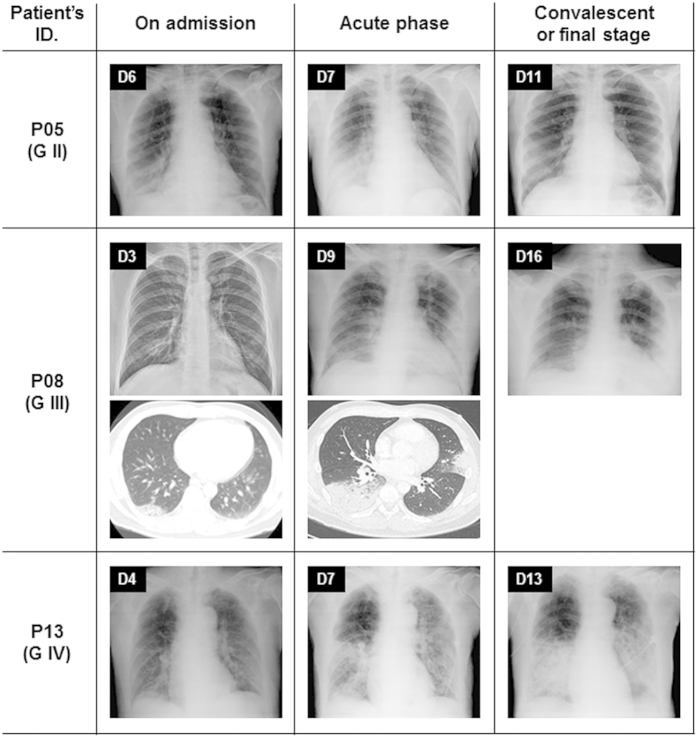
Representative chest images of the MERS patients. Chest images of the MERS patients suffered from mild (P05) or severe pneumonia (P08 and P13) were presented. P05 and P08 were recovered from the pneumonia whereas P13 died due to ARDS. Images were taken at the indicated days after symptom onset. Paired chest radiographs and computed tomography (CT) scans were performed on the same day in P08. Initial chest radiographs shows patchy consolidation along with ground-glass opacities involving unilateral or both lung zones. Follow-up frontal chest radiographs and CT scan (P08) obtained in acute phase show progression in density and extent in all three patients. Chest radiographs taken in convalescent stage show improvement in survived patients (P05 and P08) whereas that of P13 shows progression with bilateral extensive consolidation in both lung zones.

All the patients with pneumonia were treated with anti-viral drugs (Supplementary Table S2) and examined daily by chest X-rays until the resolution of pneumonia. Representative radiologic findings of MERS patents with pneumonia are shown in Fig. 1. Patients with pneumonia almost always showed ground-glass opacity or airspace consolidation involving either or both sides of lower lung fields. Chest computed tomography (CT) was performed on P08 and his chest CT scans revealed lobar consolidation in right lower lobe and segmental infiltration in the left upper lobe. Patients in group II showed more rapid clearing of pneumonic infiltrates compared to patients in group III.

### Kinetics of viral shedding from respiratory secretions and plasma

**Figure 2:**
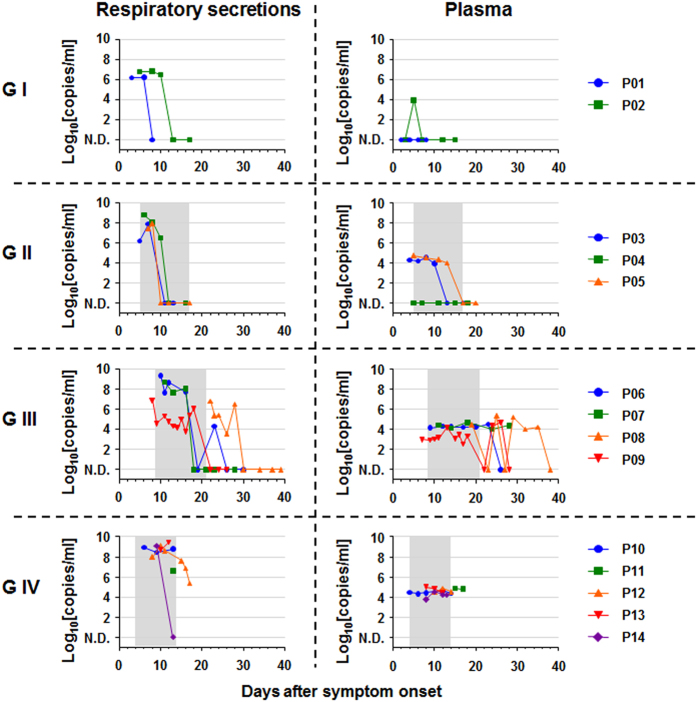
Kinetic viral loads in respiratory secretions and blood of MERS patients. Changes of average viral loads in respiratory and plasma samples are presented. The patients were classified into group I ~ IV (G I ~ IV) based on disease severity and mortality as described in the text. Gray boxes indicate the average periods of antiviral therapy administered to the patients of each group. P09 in group III received convalescent plasma therapy twice on days 10 and 16 after symptom onset.

Since the lower respiratory samples had higher viral loads and showed more sustained viral release than upper respiratory secretions^14^,^15^, we primarily measured viral loads in lower respiratory secretions such as sputa and tracheal aspirates (Fig. 2). Patient P01 secreted 1.5–1.7 × 10^6^ virus copies/ml in the sputa during the first week and P02 shed a similar level (3.1–6.7 × 10^6^ copies/ml) of virions up to ten days after fever onset. No viremia was observed in P01, but a transient viremia (8.9 × 10^3^ copies/ml) occurred in P02 on day 5. In group II patients with mild pneumonia, viral shedding (1.6 × 10^6^ to 6.5 × 10^8^ copies/ml, average = 3.5 × 10^7^ copies/ml) was detected in sputa up to ten days after symptom onset. Viremia (average = 2.2 × 10^4^ copies/ml) was observed in P03 and P05 until ten and thirteen days, respectively, whereas P04 had no viremia. Since P05 complained of severe headache and nausea from day 12 to 13^16^,^17^, cerebrospinal fluid (CSF) was collected and examined for inflammatory signs and viremia, but we detected no evidence of CNS infection. Viral shedding from group II patients stopped after initiation of antiviral therapy. In group III patients who suffered from more severe pneumonia, viral secretion in the lower respiratory tract was more sustained, continuing for up to 18 to 27 days following the onset of symptoms. In addition, viremia was detected in serum samples for up to 34 days as in the case of P08. Viral shedding ceased during antiviral therapy in two patients (P07 and P09), whereas viremia continued in all patients. Initial viral loads in respiratory secretions during the second week ranged from 3.9 × 10^4^ to 2.4 × 10^9^ copies/ml. However, the average viral load in plasma was around 1.1 × 10^4^ copies/ml, similar to group II patients. In the case of P09 who received convalescent-phase plasma twice, viral loads in respiratory secretions increased slightly and the level of viremia remained virtually unchanged until day 18. Viral loads in lower respiratory secretions from the fatal cases (group IV) were relatively higher (5.0 × 10^8^ to 1.4 × 10^9^ copies/ml, average = 6.3 × 10^8^ copies/ml) than survivors. Viremia (average = 3.2 × 10^4^ copies/ml) was also sustained during antiviral therapy in group IV cases.

### Kinetic responses of serum IgG and secretory IgA in respiratory samples

**Figure 3:**
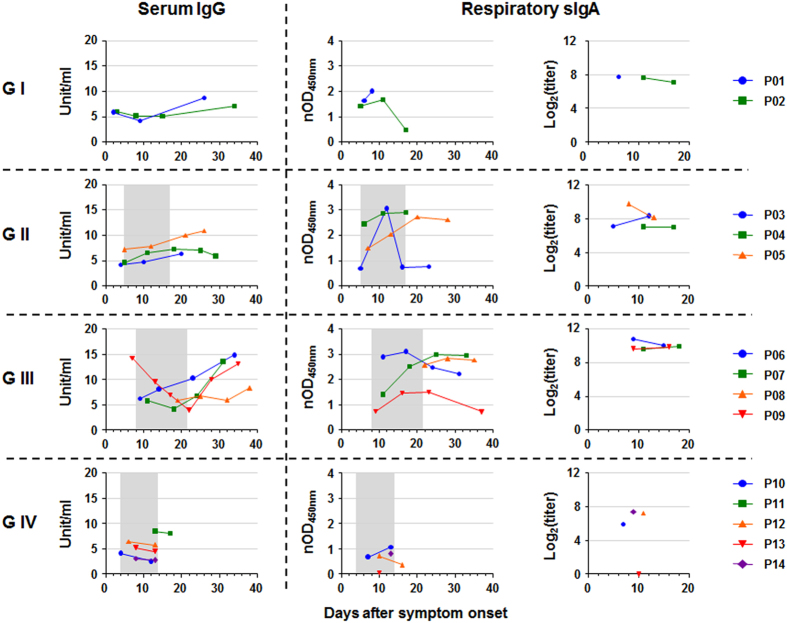
Kinetic responses of serum IgG and respiratory IgA antibody in MERS patients. Serum IgG and secretory IgA specific to spike (S1) antigen in respiratory samples were measured by ELISA. Definition of units and normalized OD (nOD) of antibody response are described in the Methods section. Gray boxes indicate the average periods of antiviral therapy administered to the patients of each group. P09 in group III received convalescent plasma therapy twice on days 10 and 16 after symptom onset.

Antibody responses against spike (S1) antigen were measured by ELISA and compared among the four groups. Gradual increases of S1-specific serum IgG were observed in all the recovered patients regardless of disease severity, whereas serum IgG responses were generally static until the second week in the lethal patients (Fig. 3). P09 who had a long incubation period (~14 days) showed decreased antibody responses after passive plasma therapy even though she showed the highest IgG response among the patients at day 7. The effect of antiviral therapy on IgG responses is not clear.

We also examined S1-specific secretory IgA responses in respiratory secretions (Fig. 3 and Supplementary Fig. S2). The level of antigen-specific secretory IgA, as measured by relative OD or titers, generally increased up to two to three weeks after symptom onset and then reached steady state or declined in all the recovered patients. Notably, secretory IgA titers of group III patients were relatively higher than mild cases during the second and third week. Among the deceased patients, three patients (P10, 12, and 14) developed detectable secretory IgA during the second or third week, whereas P13 failed to produce the specific IgA until day 10.

**Figure 4:**
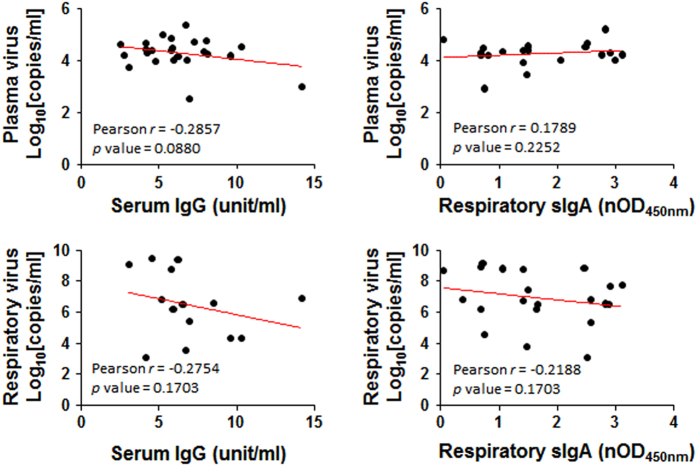
Correlation of antibody responses and viral loads. The level of antibody and viral loads in the same sample were plotted. Statistical analysis was performed by linear regression assay as described in Materials and Methods.

The levels of S1-specific antibody responses were compared against viral loads in plasma and respiratory secretions (Fig. 4). The level of serum IgG was inversely correlated with viral loads in both plasma and respiratory secretions although these did not reach statistical significance. Respiratory viral loads were also negatively correlated with secretory IgA levels without statistical significance. Secretory IgA did not show any negative correlation with viral loads in plasma. The consistently high viral loads in the clinical samples even in the presence of specific antibodies suggest that the early antibody responses induced in systemic and mucosal immune systems may not provide sufficient protective immunity against MERS-CoV replication. Nevertheless, since we measured viral nucleic acid levels rather than infectious viral titers in the clinical samples, we cannot exclude the possibility that viral infectivity is lower due to opsonization by antiviral antibodies.

### Kinetic responses of white blood cells and platelets

**Figure 5:**
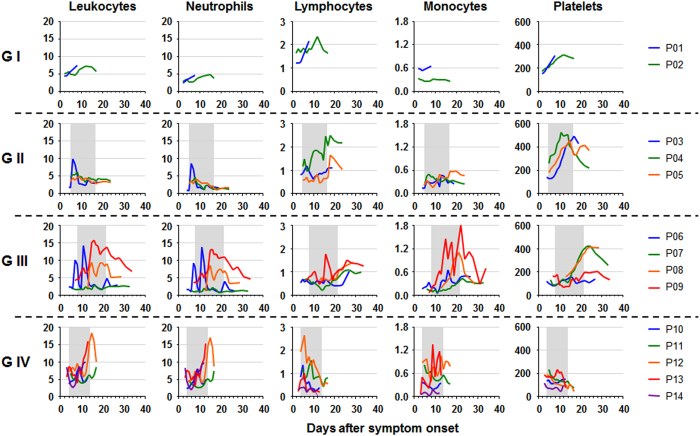
Kinetic responses of leukocyte subsets and platelets in MERS patients. The changes in absolute counts (×10^3^ cells/μL) of the indicated subset of leukocytes and platelets are presented. Gray boxes indicate the average periods of antiviral therapy administered to the patients of each group.

Hematological changes during the course of MERS were examined in all the patients (Fig. 5, Supplementary Table S3). In group I patients, there were no significant abnormalities observed in leukocytes counts and absolute counts remained within the normal range. We did note a gradual increase of lymphocytes and platelets up to 2 week after symptom onset. The gradual rise in lymphocyte and platelet counts was also observed in group II patients up to 2 ~ 3 weeks after symptom onset. P03 also showed a transient rise in neutrophil counts during the second week. Transient or persistent leukocytosis, primarily due to increased neutrophils, was observed in P06, P08, and P09 among group III patients. Monocyte counts also rose rapidly in P08 and P09 during the second and third week, but returned to basal levels thereafter. Thus, all the recovered patients exhibited a gradual increase in lymphocyte counts, suggesting that lymphocyte responses may be correlated with disease resolution. Among the five lethal cases, neutrophilia (>8,000 cell/μl) was observed in P12 and P13. Early increases of lymphocyte counts occurred in all the group IV patients except P14, however, they suffered from lymphopenia (<1,500 cells/μl) and thrombocytopenia (<140 × 10^3^/μl) during antiviral therapy. Slight increases in monocyte counts were detected in P11, P12, and P13 during treatment.

### Kinetic responses of cytokines and chemokines

**Figure 6:**
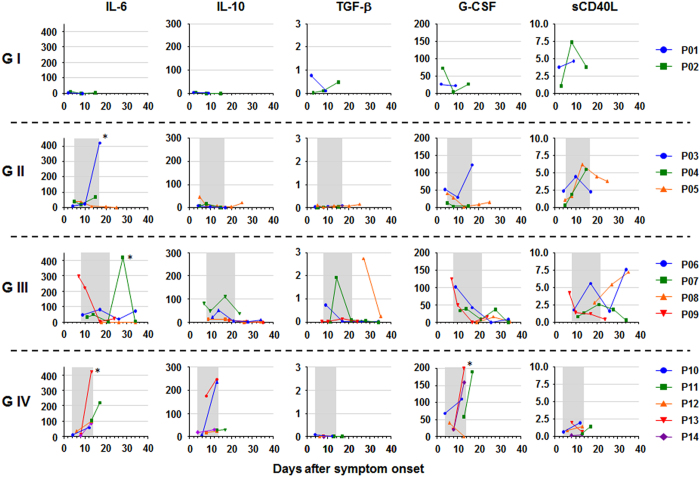
Kinetic responses of cytokines in MERS patients. Among the 39 cytokines and chemokines analyzed, kinetic changes of five representative cytokines that showed differences in the level and/or kinetics among the patients of different groups, are presented. Gray boxes indicate the average periods of antiviral therapy administered to the patients of each group. The concentrations of the cytokines are presented in pg/ml except TGF-β (ng/ml). *: data point is out of y-axis range.

**Figure 7:**
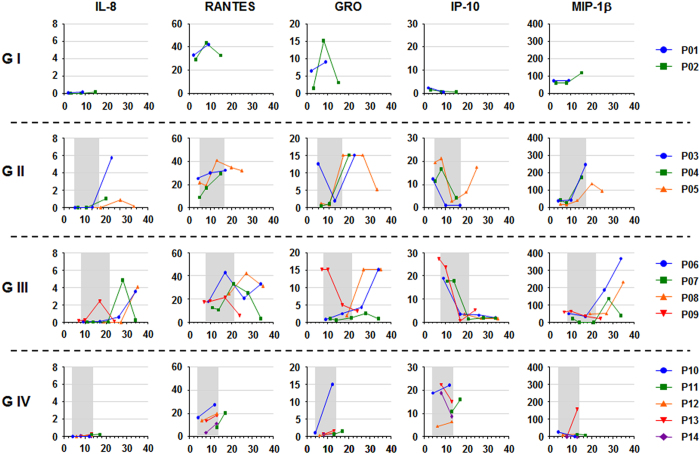
Kinetic responses of chemokines in MERS patients. Among the 39 cytokines and chemokines analyzed, kinetic changes of five representative chemokines that showed differences in the level and/or kinetics among the patients of different groups, are presented. Gray boxes indicate the average periods of antiviral therapy administered to the patients of each group. The concentrations of IL-8, GRO, and IP-10 are presented in ng/ml and those of RANTES and MIP-1β in pg/ml.

Prominent and differential changes in the white blood cells prompted us to measure the cytokines and chemokines in the patients’ plasma. We measured 39 cytokines, chemokines, and growth factors at different time points (Supplementary Table S4). IFN-α was substantially elevated in most of the patients and peaked during the second week after symptom onset (Fig. S3). Since all group II ~ IV patients received pegylated IFN-α, the elevation of the cytokines may be primarily attributed to a response to therapy. G-CSF and GM-CSF, which stimulate the bone marrow to produce granulocytes and monocytes, were also upregulated in the patients (Fig. 6. and Supplementary Fig. S4). Moreover, G-CSF rapidly increased but patients who died had relatively poor GM-CSF responses during antiviral treatment. We also measured several pro- or anti- inflammatory cytokines such as IL-1, TNF-α, IL-6, IL-10, and TGF-β in the patients’ plasma (Supplementary Table S4 and Fig. 6). Most of the patients with pneumonia had relative elevations of these cytokines during the second and third week. Some cytokines, such as IL-6 and IL-10, were generally higher in severe (group III) and fatal cases (group IV) than in mild cases (group I and II) (Fig. 6). It is also interesting to note that TGF-β responses were prominent only in group I and group III patients but barely detected in group II and group IV patients (Fig. 6). Robust induction of diverse chemokines including Fractalkine, MCP-1 (CCL2), IP-10 (CXCL10), MDC (CCL22), RANTES (CCL5), IL-8 (CXCL8), MIP-1 (CCL3), Eotaxin (CCL11), and GRO (CXCL1) were observed in most of the patients although the their levels and expression kinetics were quite variable (Supplementary Table S4 and Fig. 7). Transient upregulation of IL-8 and MCP-3 (CCL7) was more prominent in group II and III patients than group I and IV patients. IP-10 was significantly higher in pneumonia patients (group II ~IV) than group I patients. Production of IP-10 generally waned during treatment periods in patients that recovered from pneumonia (group II and III). Expression of MIP-1β was also highly induced in group II and III patients but with differential kinetics. In group II, MIP-1β was elevated during treatment periods but increased in three group III patients (P06, P07, and P08) after antiviral therapy (Fig. 7). This chemokine was barely induced in the lethal patients (group IV) except P14. Induction of GRO also showed similar kinetics as MIP-1β (Fig. 7). Robust induction of MCP-1, MDC, IP-10, and Fractalkine, which recruit monocytes and/or T cells to inflamed tissues, may contribute to inflammatory responses and/or viral clearance in the lungs. In addition, chemokines such as IL-8, MIP-1, and GRO, may help recruit neutrophils and induce the infiltration of immune cells into infected lung tissues during viral pneumonia. The induction of RANTES and Eotaxin, which recruit eosinophils and/or basophils, was detected in all the patients, suggesting a potential role of these innate immune cells in lung inflammation in MERS patients (Supplementary Table S4 and Fig. 7).

Cytokines (IL-2, IL-4, IL-5, IL-7, IL-9, IL-12, IL-15, IL-17 A, and IFN-γ) regulating T cell homeostasis and functional responses were detected only in some patients and none of the cytokines seem to be correlated with disease severity or mortality (Supplementary Table S4 and Supplementary Fig. S3). IFN-γ, a key cytokine involved in the activation of Th1 cells, was consistently observed in most patients during the first and second weeks after symptom onset. Interestingly, two cytokines, IL-7 and IL-15, regulating T cell homeostasis 18, were substantially increased in most patients. In particular, IL-15 responses were significantly higher in pneumonia patients (except P12) than group I patients (Supplementary Fig. S4).

Soluble CD40 ligand (sCD40L) was also increased in the patients’ plasma (Fig. 6). Since sCD40L has a cytokine-like activity in inflammatory responses and is primarily produced by activated platelets^19^, the level of this serum protein may reflect platelet activity during MERS progression. Indeed, the kinetic responses of sCD40L remained suppressed among group IV patients who suffered from thrombocytopenia (Fig. 5).

Several growth factors such as EGF, FGF-2, VEGF, and TGF-α were also significantly elevated in the plasma of MERS patients (Supplementary Table S4 and Supplementary Fig. S3). It is interesting to note that the level of EGF was significantly higher in the recovered patients compared to fatal cases, whereas the level of TGF-α was generally higher in the patients that suffered from severe pneumonia compared to mild cases. Previously, it was shown that EGF has a protective effect on gastrointestinal insults by porcine epidemic diarrhea virus (PEDV), a member of Coronavirus family, potentially via promoting the recovery of damaged mucosal epithelium^20^.

### Statistical analysis of virological and immunological factors associated with mortality

The hazard ratios (HR) of mortality according to the levels of various virological and immunological factors are shown in Supplementary Table S5. Overall, patients with higher antibody responses (especially respiratory IgA) and lymphocyte responses, as well as lower leukocyte and neutrophil counts, had significantly lower hazard ratios for mortality after MERS-CoV infection although the hazards ratio of white blood cell counts is very limited (IgA (nOD), HR = 0.082, 95% CI = 0.007–1.002; leukocytes counts, HR = 1.0005, 95% CI = 1.0001–1.0008; neutrophil counts, HR = 1.0005, 95% CI = 1.0001–1.0009; lymphocytes counts, HR = 0.9954, 95% CI = 0.9909–0.9999). Regarding cytokines, chemokines, and growth factors, patients with higher levels of IL-10 and IL-15 as well as lower levels of TGF-β, RANTES, and EFG had higher hazard ratios for mortality, which were marginally significant after adjusting for age (IL-10, HR = 1.013, 95% CI = 1.002–1.024; IL-15, HR = 1.146, 95% CI = 1.022–1.287; TGF-β, HR = 0.876, 95% CI = 0.770–0.998; RANTES, HR = 0.900, 95% CI = 0.780–1.015; EGF, HR = 0.9633, 95% CI = 0.9303–0.9974).

In addition to virological and immunological factors, patients’ gender and comorbidities seem to be associated with mortality and morbidity. Although the association of gender on mortality was not statistically significant (*p* = 0.071) potentially due to limited patient numbers, it is worth nothing that two-thirds of patients in the severe disease groups (group III and IV) were men and all the patients in the mild disease groups (group I and II) were women. The presence of chronic underlying illness before MERS-CoV exposure, as well as age (*p* = 0.002 tested by Log-rank test), was significantly associated with mortality. These findings are consistent with other case series as reviewed previously^1^.

## Discussion

The rapid and wide spread of MERS-CoV infection in South Korea during the outbreak had a disastrous impact on the whole country and highlights our limited knowledge on MERS. However, most Korean MERS cases reported had well documented transmission routes and exposed periods, enabling us to finely characterize disease progression in infected patients. In this study, we systemically analyzed clinical, virological, and immunological responses in a group of patients that collectively represent a broad spectrum of MERS severity, from mild febrile to fatal. Although the number of patients included in this study is limited, our data help more clearly delineate MERS progression in humans, and also provide scientific basis for better preventive measures. In addition, the observed responses of multiple factors and their association with disease severity during therapeutic periods might help us develop proper strategies to improve the efficacy of current MERS treatments.

The fourteen patients could be readily classified into four clinical groups based on disease severity and mortality. Group I patients (14.2%, 2/14) produced MERS-CoV (1.5 ~ 6.7 × 10^6^ copies/ml) in sputa up to 10 days after fever onset. Considering their long incubation periods (12 and 18 days) viral shedding may precede the onset of fever. Therefore, early diagnosis and quarantine of persons who have had contact with MERS patients may be required to prevent MERS-CoV spread, even if they are asymptomatic or have mild symptoms. Since secretory IgA specific to spike antigen increased in the respiratory tract for up to 10 days after symptom onset, MERS-CoV specific mucosal antibody responses may be a potential diagnostic biomarker in mild cases early in the disease course.

Among the patients that developed pneumonia but completely recovered from MERS (50.0%, 7/14), effective viral clearance was achieved with antiviral therapy only in group II patients (21.4%, 3/14), whereas sustained viral secretion was observed, especially in blood, in group III patients (28.6%, 4/14) even following antiviral therapy. Considering that viral shedding was poorly controlled in the fatal cases (35.7%, 5/14), it is possible that only a limited proportion of MERS patients responded to the current antiviral regimen 12. The MERS-CoV receptor, dipeptidylpeptidase 4 (DPP4), is expressed in greater abundance in the lung parenchyma (alveolar type I and II cells, macrophages and vascular endothelia) than in the upper airways and conducting airway epithelia^21^. In addition, DPP4 expression is inducible and may increase in some chronic disease conditions^21^. We speculate that the higher respiratory tract virus shedding observed in the more severe and fatal cases may reflect both the normal distribution of DPP4 and increases in receptor abundance associated with chronic disease states.

Most of the patients developed virus-specific serum IgG and secretory IgA in the respiratory tract except one fatal case (P13) who failed to develop secretory IgA. Consistent with a recent report^14^, the antibody responses in the blood as well as in the respiratory tract of survivors were more sustained and elevated than in fatal cases. Although we did not measure the neutralizing activity of the antibody responses, it was reported that the specific ELISA values are generally correlated with neutralizing antibody titers^14^,^22^. Nevertheless, viral shedding was detected in the presence of specific antibody responses, suggesting a weak protective effect against viral replication during primary MERS-CoV infection^14^. The patient who received convalescent-phase plasma (P09), showed no significant changes in blood and respiratory tract viral loads during the subsequent several days following treatment (Fig. 2). Rather, serum IgG responses rapidly dropped after plasma therapy (Fig. 3). Although we could not explain these unexpected responses, they are consistent with a report that a Korean patient given plasma therapy showed more delayed antibody responses in the days following treatment compared to other patients^22^. Although a more systematic study of patients receiving plasma therapy is needed, our findings suggest that passive antibody transfer may have limited efficacy. A previous report on plasma therapy in SARS patients showed that benefit was achieved only in a subset of patients when administered within 14 days after the onset of illness^23^.

One of the most prominent factors associated with MERS disease severity and outcome was the hematological changes in leukocyte populations (Fig. 5). Gradual and persistent increases of lymphocyte counts during MERS progression was observed in all the survivors, whereas all the deceased patients showed rapid drops of lymphocyte counts during the second week, resulting in lymphopenia. In contrast, lymphocyte counts rapidly increased up to 10 days after symptom onset in group I patients and one group II patient (P04), strongly suggesting that adaptive immune responses play a protective role. In most patients that recovered from pneumonia (groups II and III), the kinetics of lymphocyte responses were rather delayed but generally correlated with resolution of viral pneumonia. Elevation of chemokines, including MCP-1, IP-10, Fractalkine, and RANTES, which can recruit T lymphocytes, may contribute to cell-mediated antiviral responses in inflamed tissues. Among these, RANTES was rapidly elevated during the first week in group I patients and rather delayed but robustly induced in group II and III patients (Fig. 7). The chemokine was also increased in group IV patients but generally weaker than in recovered patients. Increased levels of IFN-γ (Supplementary Fig. S3) during the first and second week also support anti-viral T cell responses in the inflamed tissues^24^. Recently, it was shown that cytokines and chemokines are actively expressed in MERS-CoV-infected dendritic cells and macrophages^25^,^26^. Nevertheless, other cytokines associated with the functional activation of specific subsets of T cells were barely detected in our study. Instead, elevations of IL-7 and IL-15 with differential kinetics were noted. An association between elevations of IL-7 and IL-15 and inflammatory pulmonary diseases has been previously reported in humans^27^,^28^, as well as in MERS-CoV infected animals^29^. In addition, leukocytosis characterized by increased neutrophils and monocytes was primarily observed in several group III and IV patients, suggesting that innate immune cell responses rather than anti-viral responses might be responsible for MERS pathogenesis in the lung. Elevations in G-CSF during the second and third week, especially in fatal cases, may account for the observed leukocytosis. Although most of the chemokines involved in the recruitment of neutrophils and monocytes were highly elevated in group II and III patients who suffered from pneumonia, the overall chemokine responses, except MCP-1 and IP-10, were relatively reduced in the fatal cases. In addition, a specific correlation between chemokine levels and disease mortality was not observed. Among the inflammatory cytokines examined, TNF-α and IL-6 levels during the acute phase were generally, but not absolutely, associated with disease severity and mortality^1^, whereas the anti-inflammatory cytokines, IL-10 and TGF-β were significantly correlated with disease mortality (Supplementary Table S5). Previously, it was shown that ARDS caused by SARS-CoV infection is associated with the induction of inflammatory cytokines and chemokines such as IL-1, IL-6, IL-8, IP-10, and TNF-α, many of which were highly expressed in the lungs of SARS patients^30^. Interestingly, TGF-β responses were elevated in group I and III patients. Considering that TGF-β is involved in the antibody class switching to IgA^31^, wound healing and scar formation via fibrosis, as well as suppression of adaptive immune responses during lung inflammation^32^, the differential expression of the cytokine in MERS patients might be linked to disease severity and/or protection against the viral infection. Nevertheless, since pre-existing comorbidities, especially in group IV, likely contribute to the observed variation in cytokines and chemokines during MERS progression, we may need to be more careful in interpreting our results and the association of chemokines and cytokines with disease severity and mortality.

In conclusion, we analyzed diverse virological and immunological factors which may affect the severity, progression, and outcome of MERS in a well-defined clinical setting. High viral loads, weak antibody responses, and lymphopenia accompanied by thrombocytopenia were associated with disease mortality, whereas persistent lymphocyte responses might be required for effective immunity against MERS-CoV infection. Leukocytosis primarily due to an increase in neutrophils and monocytes was generally observed in more severe and fatal cases. The blood levels of cytokines such as IL-10, IL-15, TGF-β, and EGF were correlated with disease severity. Robust induction of various chemokines such as IL-8, IP-10, MIP-1, GRO, and RANTES with differential kinetics were more prominent in patients that recovered from pneumonia than in patients with mild febrile illness or deceased patients. Although the number of patients in this study was limited, the correlations between virus shedding and immunological responses with disease severity and mortality, as well as responses to current antiviral therapy, may indicate prognostic significance during the acute phase after symptom onset. Further studies on the functional relationship of these factors with effective immunity and pathogenesis in MERS need to be conducted.

## Methods

### Study approval

Clinical data and specimens obtained from the MERS patients were used in this study after ethnical approval granted by the institutional review boards of Chungnam National University Hospital and Seoul National University Hospital. This study was performed in accordance with the ethical standards laid down in the 1964 declaration of Helsinki and all subsequent revisions. All surviving patients provided written, informed consent to participate. In fatal cases, we obtained an exemption of patients’ consent from the institutional review boards for the retrospective analysis of clinical samples.

### Patients and study design

MERS-CoV infection was confirmed in fourteen patients treated at Chungnam National University Hospital between June 7th and August 5th, 2015 by positive real-time RT-PCR assay targeting the *upE* and *orf1a* sequences at a Korean Center for Disease Control laboratory. We reviewed clinical information and daily findings from history and physical examination, pulse oximetry, and haematological, biochemical, radiological investigations. Clinical specimens collected for viral diagnosis included respiratory secretions, blood, cerebrospinal fluids, and ascitic fluids of MERS patients. These were used for quantification of specific antibodies, subsets of cytokines, and viral loads. Pneumonia severity index (PSI) was used to estimate the severity of pneumonia in each patient as described previously^33^. We classified the patients into four groups based on disease severity and mortality, and performed comparative analysis.

### Quantitation of viral loads

Total RNA was extracted from respiratory samples, including sputa and tracheal aspirates, and plasma using TRIzol LS reagent (Thermo Fisher Scientific) according to the manufacturer’s instruction. The RNA samples were then used for titration of viral genomes using a quantitative real-time RT-PCR kit (Cosmogenetech Co. and Kogenebiotech) amplifying *orf1a*, *orf1b*, or *upE* sequences^34^. Viral copy numbers were estimated by comparing Ct values of each sample with dilutions of positive control viral cDNAs according to the manufacturer’s instruction (Cosmogenetech Co). In our hands, the detection limit of viral copy numbers was ~10^4^ copies/ml when *orf1a* and *orf1b* were targeted and ~10^3^ copies/ml when *upE* was used as the target. If triplicate results showed consistently positive reactions, the copy number data were used for kinetic analysis even if they were below detection limit. Otherwise, the data were regarded as negative.

### Quantitation of specific antibody responses

Specific antibody responses to the MERS-CoV spike protein S1 domain were measured by ELISA, using a recombinant MERS-CoV spike protein S1 domain control^35^, according to the manufacturer’s instruction (Anti-MERS-CoV S1 ELISA kit, Alpha Diagnostic International). Specific IgG responses were determined as unit/ml by comparing the net O.D. value of each plasma sample with calibration standards assayed together in each experimental set (Alpha Diagnostic International). Secretory IgA responses were also semi-quantitated and titrated using serial dilutions of the respiratory samples using the same ELISA kit, with substituting an anti-human IgA-horseradish peroxidase conjugate (SouthernBiotech) as the secondary antibody^36^. Since lower respiratory tract samples such as sputa were difficult to analyze directly, they were processed before performing ELISA as described previously^37^. Due to limited samples, antibody responses were measured at 3 ~7 days interval and all the data obtained were included for kinetic analysis.

### Quantitation of cytokines and chemokines

Thirty eight cytokines, chemokines, and growth factors were measured in plasma samples using MILLIPLEX MAP 38-plex cytokine assay kit (Millipore) as specified by the manufacturer’s instructions. These included sCD40L, VEGF, TNF-β, TNF-α, TGF-α, MIP-1β, MIP-1α, MDC (CCL22), MCP-3, MCP-1, IP-10, IL-17, IL-15, IL-13, IL-12 (p70), IL-12 (p40), IL-10, IL-9, IL-8, IL-7, IL-6, IL-5, IL-4, IL-3, IL-2, IL-1ra, IL-1β, IL-1α, IFN-γ, IFN-α2, GRO, GM-CSF, G-CSF, Fractalkine, Flt-3 L, FGF-2, Eotaxin, and EGF. RANTES was measured independently using a quantitative ELISA kit (BioLegend). All the samples were measured in duplicate and the mean values were used for analysis.

### Statistics

All the data from each patient were arranged from date of exposure to an infected patient to the date of death (event, n = 5) or to the date that recovered patients were discharged from the hospital (censored case, n = 9) (Supplementary Fig. S1). We performed time-varying analyses of each data value obtained from the patients’ samples. There were total 67 to 103 individual observations, depending on the number of measurements of virological and immunological factors, during 461 observation period. Cox-proportional hazards regression models with the virological and immunological factors entered as time varying covariates were used to calculate crude hazard ratios (HRs) with 95% confidence intervals (CIs) of the factors per 1 unit of mortality. All the factors were treated as continuous variables. Age-adjusted hazard ratios were calculated for every factor. In this study, a *p*-value less than 0.05 (two-tailed) was considered statistically significant. All statistical analyses were performed by Stata (version 12.1; Stata Co.). Statistical analyses of the correlation of antibody responses and viral loads was performed by linear regression assay embedded in GraphPad Prism software (Ver. 5.0).

## Additional Information

**How to cite this article**: Min, C.-K. *et al*. Comparative and kinetic analysis of viral shedding and immunological responses in MERS patients representing a broad spectrum of disease severity. *Sci. Rep*. **6**, 25359; doi: 10.1038/srep25359 (2016).

## Supplementary Material

Supplementary Information

## Figures and Tables

**Table 1:** Clinical characteristics of MERS patients included in this study.

Group		Patient’s ID.	Age	Sex	Underlying diseases*	Incubation period (days)	Fever onset to pneumonia (days)	Severity of Pneumonia (PSI)**	Anti-viral therapy (days)***	Defervescence after the therapy (days)****	O_2_ therapy (days)*****
Recovered	G I	P01	65	F	–	18	–	–	–	–	–
		P02	66	F	–	12	–	–	–	–	–
	G II	P03	39	F	–	8	3	29	12	3	–
		P04	55	F	–	10	4	45	14	5	–
		P05	57	F	–	9	4	47	14	9	–
	G III	P06	60	M	–	5	4	60	19	19	–
		P07	63	F	–	6	5	63	14	10	NP (1)
		P08	41	M	FAP	4	2	71	7	2	NP (1)
		P09	61	F	–	14	6	71	14	10	MV/ECMO (14/7)
Deceased	G IV	P10	82	F	–	5	7	102	11	N.D.	HFNC (3)
		P11	49	M	Cirrhosis, DM	4	7	119	10	N.D.	HFNC (2)
		P12	69	M	TB	3	5	119	11	N.D.	MV (5)
		P13	74	M	COPD	2	2	144	11	N.D.	MV (7)
		P14	78	M	COPD, Asthma, DM	2	3	148	10	4	MV (4)

^*^FAP: Familial adenomatous polyposis, DM: Diabetes mellitus, COPD: Chronic obstructive pulmonary disease, TB: Tuberculosis.

^**^PSI: Pneumonia severity index at initial presentation.

^***^Anti–viral therapy: combined therapy composed of pegylated interferon (IFN)-alpha 2a, ribavirin, and lopinavir/ritonavir (see details in Supplementary Table S2).

^****^N.D.: No defervescence.

^*****^NP: nasal prong, HFNC: high flow nasal cannula, MV: mechanical ventilation, ECMO: extracorporeal membrane oxygenation.

## References

[b1] Chan J. F. . Middle East respiratory syndrome coronavirus: another zoonotic betacoronavirus causing SARS-like disease. Clin Microbiol Rev 28, 465–522, doi: 10.1128/CMR.00102-14 (2015). 25810418 PMC4402954

[b2] WHO. *Middle East respiratory syndrome coronavirus (MERS-CoV)*, < http://www.who.int/emergencies/mers-cov/en/ >(2015) (Date of acess:31/03/2016).

[b3] Azhar E. I. . Evidence for camel-to-human transmission of MERS coronavirus. N Engl J Med 370, 2499–2505, doi: 10.1056/NEJMoa1401505 (2014). 24896817

[b4] Drosten C. . Transmission of MERS-coronavirus in household contacts. N Engl J Med 371, 828–835, doi: 10.1056/NEJMoa1405858 (2014). 25162889

[b5] Oboho I. K. . 2014 MERS-CoV outbreak in Jeddah–a link to health care facilities. N Engl J Med 372, 846–854, doi: 10.1056/NEJMoa1408636 (2015). 25714162 PMC5710730

[b6] Korea Centers for Disease, C. & Prevention. Middle East Respiratory Syndrome Coronavirus Outbreak in the Republic of Korea, 2015. *Osong Public Health Res Perspect* **6**, 269-278, doi: 10.1016/j.phrp.2015.08.006 (2015). 10.1016/j.phrp.2015.08.006 PMC4588443 26473095

[b7] Butler D. South Korean MERS outbreak spotlights lack of research. Nature 522, 139–140, doi: 10.1038/522139a (2015). 26062490

[b8] Assiri A. . Epidemiological, demographic, and clinical characteristics of 47 cases of Middle East respiratory syndrome coronavirus disease from Saudi Arabia: a descriptive study. Lancet Infect Dis 13, 752–761, doi: 10.1016/S1473-3099(13)70204-4 (2013). 23891402 PMC7185445

[b9] Feikin D. R. . Association of Higher MERS-CoV Virus Load with Severe Disease and Death, Saudi Arabia, 2014. Emerg Infect Dis 21, 2029–2035, doi: 10.3201/eid2111.150764 (2015). 26488195 PMC4622256

[b10] Memish Z. A., Zumla A. I., Al-Hakeem R. F., Al-Rabeeah A. A. & Stephens G. M. Family cluster of Middle East respiratory syndrome coronavirus infections. N Engl J Med 368, 2487–2494, doi: 10.1056/NEJMoa1303729 (2013). 23718156

[b11] Yang J. S. . Middle East Respiratory Syndrome in 3 Persons, South Korea, 2015. Emerg Infect Dis 21, 2084–2087, doi: 10.3201/eid2111.151016 (2015). 26488745 PMC4622265

[b12] Omrani A. S. . Ribavirin and interferon alfa-2a for severe Middle East respiratory syndrome coronavirus infection: a retrospective cohort study. Lancet Infect Dis 14, 1090–1095, doi: 10.1016/S1473-3099(14)70920-X (2014). 25278221 PMC7106357

[b13] Spanakis N. . Virological and serological analysis of a recent Middle East respiratory syndrome coronavirus infection case on a triple combination antiviral regimen. Int J Antimicrob Agents 44, 528–532, doi: 10.1016/j.ijantimicag.2014.07.026 (2014). 25288266 PMC7127532

[b14] Corman V. M. . Viral shedding and antibody response in 37 patients with MERS-coronavirus infection. Clin Infect Dis, doi: 10.1093/cid/civ951 (2015). PMC7108065 26565003

[b15] Memish Z. A. . Respiratory tract samples, viral load, and genome fraction yield in patients with Middle East respiratory syndrome. J Infect Dis 210, 1590–1594, doi: 10.1093/infdis/jiu292 (2014). 24837403 PMC7107391

[b16] Hung E. C. . Detection of SARS coronavirus RNA in the cerebrospinal fluid of a patient with severe acute respiratory syndrome. Clin chem 49, 2108–2109, doi: 10.1373/clinchem.2003.025437 (2003). 14633896 PMC7108123

[b17] Lau K. K. . Possible central nervous system infection by SARS coronavirus. Emerg Infect Dis 10, 342–344, doi: 10.3201/eid1002.030638 (2004). 15030709 PMC3322928

[b18] Rochman Y., Spolski R. & Leonard W. J. New insights into the regulation of T cells by gamma(c) family cytokines. Nature Rev Immunol 9, 480–490, doi: 10.1038/nri2580 (2009). 19543225 PMC2814538

[b19] Aloui C. . The signaling role of CD40 ligand in platelet biology and in platelet component transfusion. Int J Mol Sci 15, 22342–22364, doi: 10.3390/ijms151222342 (2014). 25479079 PMC4284712

[b20] Jung K. . Effects of epidermal growth factor on atrophic enteritis in piglets induced by experimental porcine epidemic diarrhoea virus. Vet J 177, 231–235, doi: 10.1016/j.tvjl.2007.04.018 (2008). 17574457 PMC7129753

[b21] Meyerholz D. K., Lambertz A. M. & McCray P. B. Jr. Dipeptidyl Peptidase 4 Distribution in the Human Respiratory Tract: Implications for the Middle East Respiratory Syndrome. Am J Pathol 186, 78–86, doi: 10.1016/j.ajpath.2015.09.014 (2016). 26597880 PMC4715219

[b22] Park W. B. . Kinetics of Serologic Responses to MERS Coronavirus Infection in Humans, South Korea. Emerg Infect Dis 21, 2186–2189, doi: 10.3201/eid2112.151421 (2015). 26583829 PMC4672454

[b23] Cheng Y. . Use of convalescent plasma therapy in SARS patients in Hong Kong. Eur J Clin Microbiol 24, 44–46, doi: 10.1007/s10096-004-1271-9 (2005). PMC7088355 15616839

[b24] Faure E. . Distinct immune response in two MERS-CoV-infected patients: can we go from bench to bedside? PLos ONE 9, e88716, doi: 10.1371/journal.pone.0088716 (2014). 24551142 PMC3925152

[b25] Zhou J. . Active replication of Middle East respiratory syndrome coronavirus and aberrant induction of inflammatory cytokines and chemokines in human macrophages: implications for pathogenesis. J Infect Dis 209, 1331–1342, doi: 10.1093/infdis/jit504 (2014). 24065148 PMC7107356

[b26] Chu H. . Productive replication of Middle East respiratory syndrome coronavirus in monocyte-derived dendritic cells modulates innate immune response. Virol 454-455, 197–205, doi: 10.1016/j.virol.2014.02.018 (2014). PMC7111975 24725946

[b27] Muro S. . Expression of IL-15 in inflammatory pulmonary diseases. J Allergy Clin Immunol 108, 970–975, doi: 10.1067/mai.2001.119556 (2001). 11742275

[b28] Yadava K. . TSLP promotes influenza-specific CD8 + T-cell responses by augmenting local inflammatory dendritic cell function. Mucosal Immunol 6, 83–92, doi: 10.1038/mi.2012.50 (2013). 22806096 PMC3534170

[b29] de Wit E. . Middle East respiratory syndrome coronavirus (MERS-CoV) causes transient lower respiratory tract infection in rhesus macaques. Proc Natl Acad Sci USA 110, 16598–16603, doi: 10.1073/pnas.1310744110 (2013). 24062443 PMC3799368

[b30] Totura A. L. & Baric R. S. SARS coronavirus pathogenesis: host innate immune responses and viral antagonism of interferon. Curr Opin Virol 2, 264–275, doi: 10.1016/j.coviro.2012.04.004 (2012). 22572391 PMC7102726

[b31] Cazac B. B. & Roes J. TGF-beta receptor controls B cell responsiveness and induction of IgA *in vivo*. Immunity 13, 443–451 (2000). 11070163 10.1016/s1074-7613(00)00044-3

[b32] Moldoveanu B. . Inflammatory mechanisms in the lung. J Inflamm Res 2, 1–11 (2009). 22096348 PMC3218724

[b33] Gilbert K. & Fine M. J. Assessing prognosis and predicting patient outcomes in community-acquired pneumonia. Semin Respir Infect 9, 140–152 (1994). 7831536

[b34] Pas S. D. . First international external quality assessment of molecular diagnostics for Mers-CoV. J Clin Virol 69, 81–85, doi: 10.1016/j.jcv.2015.05.022 (2015). 26209385 PMC7106520

[b35] Raj V. S. . Dipeptidyl peptidase 4 is a functional receptor for the emerging human coronavirus-EMC. Nature 495, 251–254, doi: 10.1038/nature12005 (2013). 23486063 PMC7095326

[b36] Muth D. . Infectious Middle East Respiratory Syndrome Coronavirus Excretion and Serotype Variability Based on Live Virus Isolates from Patients in Saudi Arabia. J Clin Microbiol 53, 2951–2955, doi: 10.1128/JCM.01368-15 (2015). 26157150 PMC4540943

[b37] Willis V. C. . Sputum Autoantibodies in Patients With Established Rheumatoid Arthritis and Subjects at Risk of Future Clinically Apparent Disease. Arthritis Rheum-Us 65, 2545–2554, doi: 10.1002/art.38066 (2013). PMC4066465 23817979

