# Supplementary material for: Comparative and kinetic analysis of viral shedding and immunological responses in MERS patients representing a broad spectrum of disease severity
Source: Sci Rep. 2016 May 5;6:25359. doi: 10.1038/srep25359 (PMC4857172; doi:10.1038/srep25359)
Supplement: Supplementary Information [file srep25359-s1.pdf]

Supplementary Data for

Comparative and kinetic analysis of viral shedding and immunological responses in MERS patients representing a broad spectrum of disease severity

Chan-Ki Min[1,2,*], Shinhye Cheon[3,*], Na-Young Ha[1,2,*], Kyung Mok Sohn[3,*], Yuri Kim[1,2], Abdimadiyeva Aigerim[1,2], Hyun Mu Shin[1,2], Ji-Yeob Choi[2,4], Kyung-Soo Inn[5], Jin-Hwan Kim[6], Jae Young Moon[7], Myung-Sik Choi[1], Nam-Hyuk Cho[1,2,8,†] and Yeon-Sook Kim[3,†]

[1]Department of Microbiology and Immunology, Seoul National University College of Medicine, Seoul, Republic of Korea. [2]Department of Biomedical Sciences, Seoul National University College of Medicine, Seoul, Republic of Korea. [3]Division of Infectious Diseases, Department of Internal Medicine, Chungnam National University School of Medicine, Daejeon, Republic of Korea. [4]Cancer Research Institute, Seoul National University College of Medicine, Seoul, Republic of Korea, [5]Department of Pharmaceutical Science, College of Pharmacy, Kyung Hee University, Seoul, Republic of Korea, [6]Department of Radiology, Chungnam National University School of Medicine, Daejeon, Republic of Korea. [7]Division of Pulmonology and Critical Care Medicine, Department of Internal Medicine, Chungnam National University School of Medicine, Daejeon, Republic of Korea. [8]Institute of Endemic Disease, Seoul National University Medical Research Center and Bundang Hospital, Seoul, Republic of Korea.

Supplementary Fig. S1. Schematic diagram of epidemiological and clinical courses of MERS patients included in this study.

Supplementary Fig. S2. Determination of antibody titers of secretory IgA in respiratory samples. Since there were no negative samples, cut-off values (mean optical densities ± 3 x S.D.) were determined by using the OD values of baselines (> 1: 6400 diluted samples).

Supplementary Fig. S3. Kinetic responses of cytokines in MERS patients. Kinetic responses of cytokines not included in Fig. 6 and showed significant upregulation in any of the clinical groups are presented. All these data values are included in Supplementary Table 3. The concentrations of the cytokines are presented in pg/ml. *: data point is out of x-axis range.

Supplementary Table S1. Laboratory data for fourteen MERS patients included in this study. Summary of data from each patient on admission and at acute phase.

Supplementary Table S2. Summary of anti-viral therapy applied to MERS patients.

Supplementary Table S3. Kinetic changes in leukocyte and platelet counts in MERS patients.

Supplementary Table S4. Kinetic responses of 39 cytokines, chemokines, and growth factors in MERS patients.

Supplementary Table S5. Hazards ratios and 95% confidence intervals of mortality according to the virological and immunological factors.

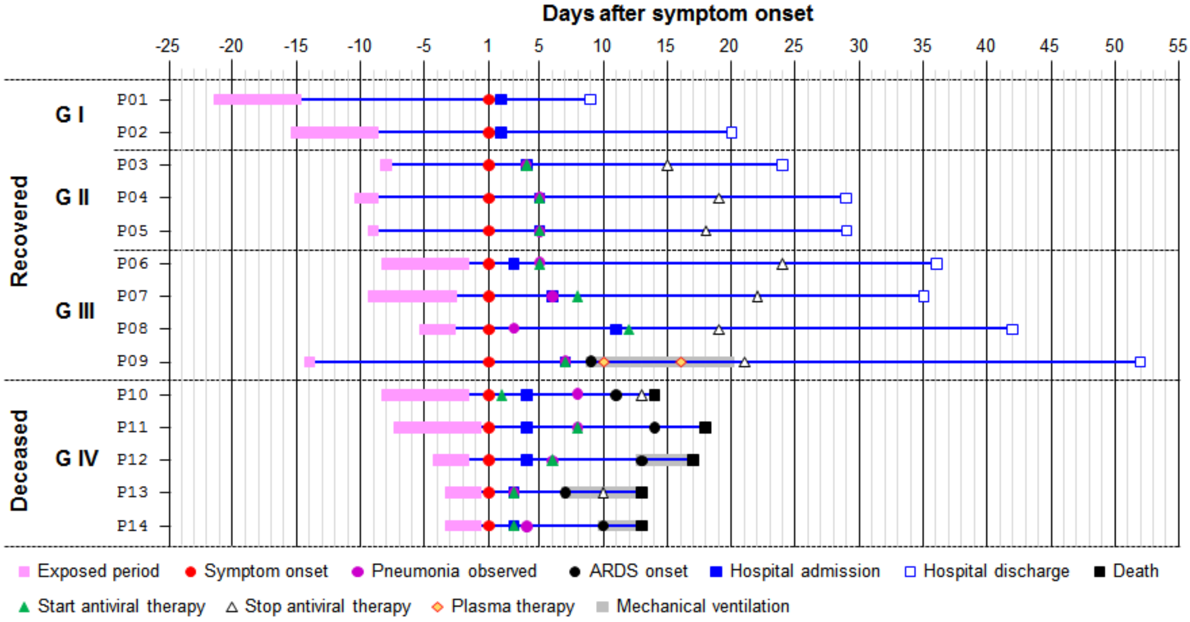

Supplementary Fig. S1. Schematic diagram of epidemiological and clinical courses of MERS patients included in this study.

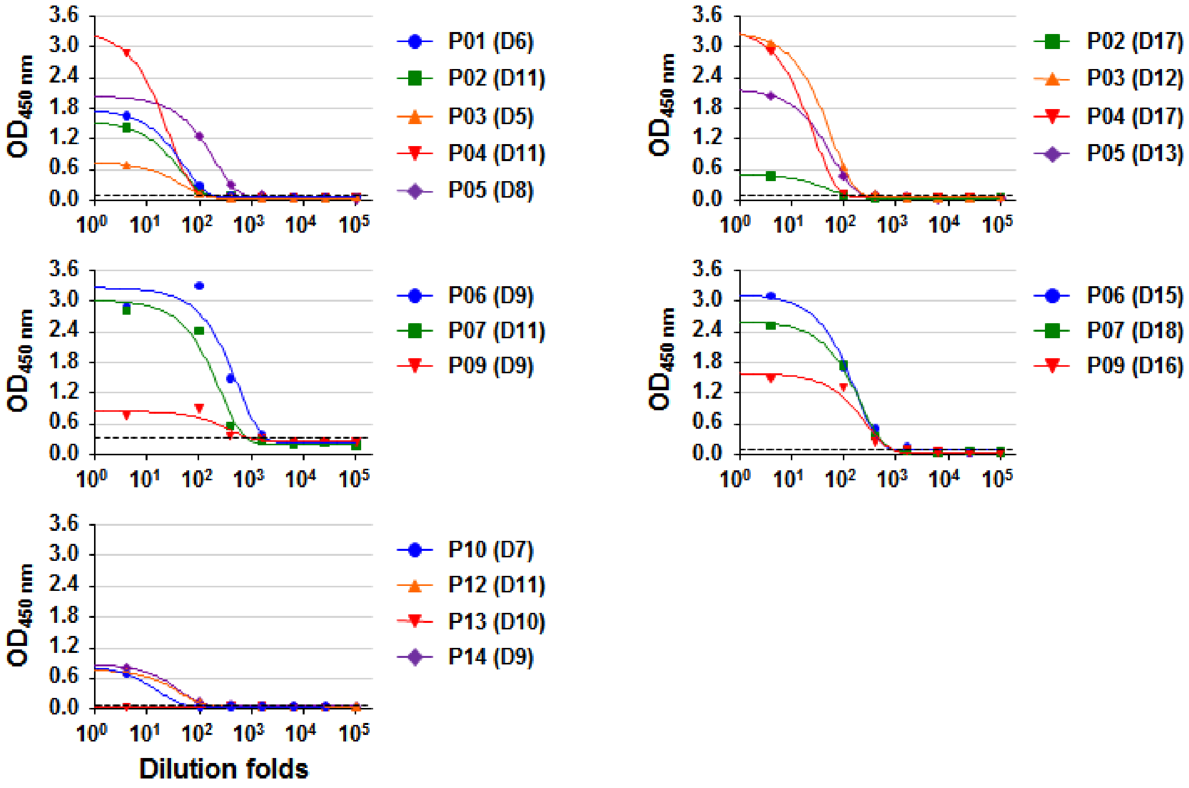

Supplementary Fig. S2. Determination of antibody titers of secretory IgA in respiratory samples. Since there were no negative samples, cut-off values (mean optical densities ± 3 x S.D.) were determined by using the OD values of baselines (> 1: 6400 diluted samples).

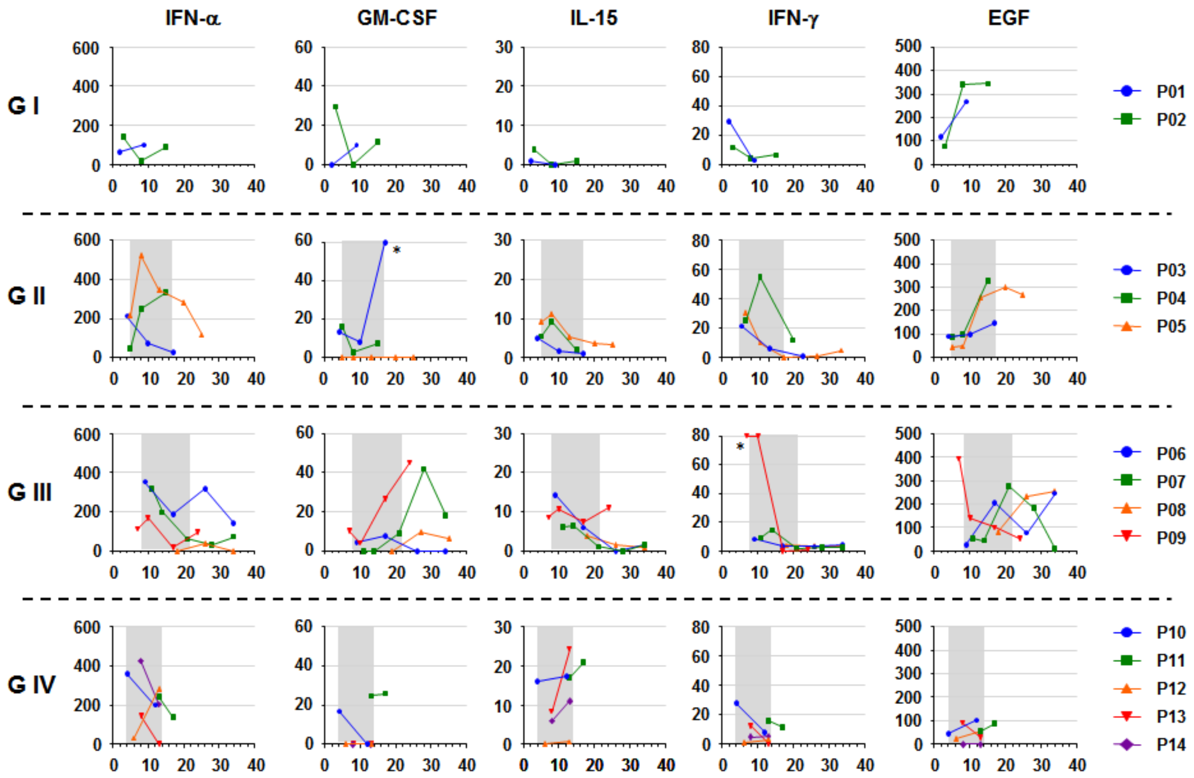

Supplementary Fig. S3. Kinetic responses of cytokines in MERS patients. Kinetic responses of cytokines not included in Fig. 6 and showed significant upregulation in any of the clinical groups are presented. All these data values are included in Supplementary Table 3. The concentrations of the cytokines are presented in pg/ml. *: data point is out of y-axis range.

Supplementary Table S1. Laboratory data for fourteen MERS patients included in this study. Summary of data from each patient on admission and at acute phase.

Group	Group I			Group II				Group III					Group IV						
Patient's ID.	P01	P02	Average	P03	P04	P05	Average	P06	P07	P08	P09	Average	P10	P11	P12	P13	P14	Average	
**On admission**																			Normal range
Days after symptom onset	D2	D2		D4	D5	D5		D3	D6	D12	D7		D4	D5	D4	D3	D3		
Leucocyte (cells/μL)	4200	4900	4550	1800	4700	3400	3300	2240	2000	1910	4100	2563	3600	6740	7000	5500	8400	6248	3800 ~ 10000
Lymphocyte (cells/μL)	1200	1600	1400	800	1200	600	867	510	600	630	600	585	800	1190	1900	200	300	878	1000 ~ 4000
Lymphocyte (%)	28.6	32.7	30.6	44.4	25.5	17.6	29.2	22.8	30.0	33.0	14.6	25.1	22.2	17.6	27.1	3.6	3.5	14.8	20 ~ 51
Platelet (cells x 10^3/μL)	145	166	156	140	257	175	191	118	131	155	165	142	135	233	184	189	111	170	150 ~ 450
Hemoglobin (g/L)	138	127	133	136	117	126	126	129	120	132	115	124	128	121	143	120	120	126	135 ~ 175
BUN (mg/dl)	10	18	14	5	8	5	6	9	12	12	13	12	11	17	9	20	21	16	8 ~ 20
Cr (mg/dl)	0.66	0.56	0.61	0.45	0.49	0.56	0.50	0.67	0.67	0.77	0.56	0.67	0.88	0.97	0.68	1.16	1.19	0.98	0.8 ~ 1.2
Albumin (g/dL)	3.6	3.5	3.6	3.9	3.1	3.1	3.4	3.3	3.5	3.1	2.6	3.1	3.4	2.3	2.6	2.8	2.5	2.7	4.0 ~ 5.0
CRP (mg/dl)	0.5	2.7	1.6	0.5	15.1	4.7	6.8	3.6	0.7	1.5	16.9	5.7	0.5	3.4	0.7	9.1	17.2	6.2	0 ~ 0.5
Procalcitonin (ng/mL)	-	-		0.05	-	-	0.05	0.05	-	0.34	0.89	0.43	-	0.74	0.07	-	0.52	0.44	0 ~ 0.05
AST (IU/L)	27	13	20	16	25	105	49	42	52	231	31	89	27	170	41	16	21	55	0 ~ 37
ALT (IU/L)	13	12	13	10	16	68	31	16	25	129	15	46	12	28	29	10	16	19	0 ~ 41
LDH (IU/L)	691	270	481	523	468	705	565	430	613	1587	530	790	384	368	328	419	452	390	200 ~ 400
Amylase (IU/L)	43	62	53	38	33	74	48	60	66	754	59	235	68	66	78	136	53	80	30 ~ 110
Lipase (IU/L)	35	26	31	34	28	41	34	27	28	967	37	265	42	40	35	29	19	33	22 ~ 51
Proteinuria	-	trace		-	1+	1+		-	trace	3+	-		trace	-	-	-	1+		
Arterial blood gas values																			
pH	ND*	ND		ND	7.40	ND	7.40	7.40	ND	7.50	7.42	7.44	ND	7.40	7.40	7.34	7.43	7.39	7.35 ~ 7.45
pCO_2 (mmHg)	ND	ND		ND	36.0	ND	36.0	40.0	ND	26.0	28.0	31.3	ND	38.0	33.0	30.0	26.0	31.8	32.0 ~ 45.0
pO_2 (mmHg)	ND	ND		ND	73.0	ND	73.0	92.0	ND	96.0	68.0	85.3	ND	87.0	74.0	97.0	56.0	78.5	75.0 ~ 100.0
HCO_3 (mmol/L)	ND	ND		ND	22.3	ND	22.3	24.8	ND	20.3	18.2	21.1	ND	24.0	21.9	16.6	17.3	20.0	19 ~ 24
O_2 saturation (%)	ND	ND		ND	94.0	ND	94.0	97.0	ND	98.0	94.0	96.3	ND	97.0	95.0	97.0	95.0	96.0	96 ~ 97
**Acute phase**																			
Days after symptom onset				D5	D8	D8		D6	D18	D12	D9		D13	D18	D17	D13	D13		
Leucocyte (cells/μL)	ND	ND		1600	5300	3700	3533	3000	1600	1400	8140	3535	5600	13600	11700	15800	9800	11300	
Lymphocyte (cells/μL)	ND	ND		600	900	400	633	500	200	500	400	400	200	400	500	200	100	280	
Lymphocyte (%)	ND	ND		37.5	17.0	10.8	21.8	16.7	12.5	35.7	4.9	17.4	3.6	2.9	4.3	1.3	1.0	2.6	
Platelet (cells x 10^3/μL)	ND	ND		140	322	228	230	88	81	163	346	170	146	66	41	74	94	84	
Hemoglobin (g/L)	ND	ND		136	114	116	122	131	114	134	114	123	128	113	130	126	115	122	
BUN (mg/dl)	ND	ND		5	6	6	6	11	8	12	6	9	14	51	21	58	61	41	
Cr (mg/dl)	ND	ND		0.45	0.49	0.58	0.51	0.68	0.60	0.65	0.57	0.63	0.73	1.34	0.60	1.00	1.51	1.04	
Albumin (g/dL)	ND	ND		3.8	3.1	2.8	3.2	2.7	3.0	3.1	3.0	3.0	2.3	2.8	2.1	2.5	2.9	2.5	
CRP (mg/dl)	ND	ND		0.5	9.6	9.8	6.6	2.1	1.2	1.0	8.1	3.1	12.9	13.4	6.7	10.2	5.5	9.7	
Procalcitonin (ng/mL)	ND	ND		-	-	-		-	-	-	56	56	-	-	-	-	-		
AST (IU/L)	ND	ND		14	17	79	37	141	49	242	79	128	110	77	39	88	35	70	
ALT (IU/L)	ND	ND		11	14	55	27	74	30	137	90	83	67	14	13	60	31	37	
LDH (IU/L)	ND	ND		384	448	786	539	1076	400	1844	596	979	1003	1054	820	1241	776	979	
Amylase (IU/L)	ND	ND		41	37	108	62	53	59	715	76	226	98	279	36	567	393	275	
Lipase (IU/L)	ND	ND		42	32	69	48	36	32	844	48	240	41	179	27	338	19	121	
Proteinuria	-	-		-	-	1+		3+	1+	3+	2+		1+	2+	2+	1+	1+		
Arterial blood gas values																			
pH	ND	ND		ND	ND	7.41	7.41	7.41	ND	7.50	7.43	7.45	7.45	7.09	7.43	7.08	7.18	7.25	
pCO_2 (mmHg)	ND	ND		ND	ND	33.0	33.0	23.3	ND	26.0	13.0	20.8	29.0	67.0	58.0	95.0	59.0	61.6	
pO_2 (mmHg)	ND	ND		ND	ND	75.0	75.0	84.0	ND	96.0	71.0	83.7	42.0	46.0	85.0	43.0	35.0	50.2	
HCO_3 (mmol/L)	ND	ND		ND	ND	21.9	21.9	22.2	ND	20.3	19.5	20.7	20.2	20.3	38.5	28.2	22.0	25.8	
O_2 saturation (%)	ND	ND		ND	ND	95.0	95.0	96.0	ND	98.0	95.0	96.3	80.0	63.0	97.0	57.0	50.0	69.4	

Supplementary Table S2. Summary of anti-viral therapy applied to MERS patients.

Anti-viral Therapy**	Patient's ID.													
	P01	P02	P03	P04	P05	P06	P07	P08	P09	P10	P11	P12	P13	P14
Pegylated IFN-α	-	-	D4*, D11	D6, D13	D5, D12	D5, D12, D19	D8, D15	D12, D19	D7, D14	D9	D8, D15	D6, D13	D3, D10	D4, D10
Ribavirin	-	-	D4 ~ D15	D6 ~ D18	D5 ~ D18	D5 ~ D24	D8 ~ D21	D12 ~ D19	D7 ~ D13	D4 ~ D13	D6 ~ D17	D6 ~ D17	D3 ~ D7	D3 ~ D13
lopinavir/ritonavir	-	-	D4 ~ D15	D6 ~ D18	D5 ~ D18	-	D8 ~ D13	-	D7 ~ D22	D4 ~ D13	D8 ~ D17	D6 ~ D17	D8 ~ D13	D8 ~ D13
Anti-viral plasma***	-	-	-	-	-	-	-	-	D10, D16	-	-	-	-	-

* Days after symptom onset.
** IFN-α2a (180 μg/week); Ribavirin (400 – 1200 mg, three times a day); lopinavir/ritonavir (400/100 mg PO every 12 h)
*** anti-S1 IgG level: 3.97 unit/ml

Supplementary Table S3. Kinetic changes in leukocyte and platelet counts in MERS patients.

Patient's ID.																					
P01	Days after symptom onset	2	3	4	8									Normal range							
	Hb (g/dl)	13.8	13.5	14.3	14.1									12 ~ 14							
	PLT (x 10^3/μl)	145	159	195	305									130 ~ 400							
	WBC (/μl)	4200	4300	5100	7300									4000 ~ 10000							
	neutrophil (/μl)	2356	2498	3284	4424									1420 ~ 6340							
	lymphocyte (/μl)	1193	1195	1244	2132									710 ~ 4530							
	monocyte (/μl)	592	559	520	642									140 ~ 720							
	neutrophils (%)	56.1	58.1	64.4	60.6																
	lymphocytes (%)	28.4	27.8	24.4	29.2																
	monocytes (%)	14.1	13	10.2	8.8																
P02	Days after symptom onset	2	3	4	5	6	7	8	9	12	15	17									
	Hb (g/dl)	12.7	13.4	13	12.9	12	11.8	12.1	12	13.5	12.6	12.1									
	PLT (x 10^3/μl)	166	188	201	203	223	231	262	280	312	294	280									
	WBC (/μl)	4900	5200	5500	4700	4800	4500	5200	6000	7120	7000	5600									
	neutrophil (/μl)	2818	2980	3427	2491	2554	2448	2881	3600	4286	4767	3517									
	lymphocyte (/μl)	1632	1804	1639	1814	1829	1625	1830	1794	2342	1736	1635									
	monocyte (/μl)	328	281	297	244	245	239	265	312	292	280	241									
	neutrophils (%)	57.5	57.3	62.3	53	53.2	54.4	55.4	60	60.2	68.1	62.8									
	lymphocytes (%)	33.3	34.7	29.8	38.6	38.1	36.1	35.2	29.9	32.9	24.8	29.2									
	monocytes (%)	6.7	5.4	5.4	5.2	5.1	5.3	5.1	5.2	4.1	4	4.3									
P03	Days after symptom onset	4	5	6	7	8	9	10	11	12	13	14	15	16	17	19					
	Hb (g/dl)	13.6	13.8	13.6	13.3	13.1	13.4	12.9	12.4	12.4	12.1	12.8	12.9	12.3	12.7	11.7					
	PLT (x 10^3/μl)	140	128	123	134	160	201	230	278	331	399	427	440	456	493	428					
	WBC (/μl)	1800	1600	9900	8220	5300	2700	2700	2400	2200	3700	3500	2900	2800	3200	3000					
	neutrophil (/μl)	839	611	8583	6732	4060	1658	1787	1274	1210	2279	2170	1572	1590	1776	1626					
	lymphocyte (/μl)	803	846	1000	1192	954	772	581	732	755	858	816	902	893	1085	1092					
	monocyte (/μl)	148	130	317	288	265	251	302	346	165	463	448	377	269	310	243					
	neutrophils (%)	46.6	38.2	86.7	81.9	76.6	61.4	66.2	53.1	55	61.6	62	54.2	56.8	55.5	54.2					
	lymphocytes (%)	44.6	52.9	10.1	14.5	18	28.6	21.5	30.5	34.3	23.2	23.3	31.1	31.9	33.9	36.4					
	monocytes (%)	8.2	8.1	3.2	3.5	5	9.3	11.2	14.4	7.5	12.5	12.8	13	9.6	9.7	8.1					
P04	Days after symptom onset	5	6	7	8	9	10	11	12	13	14	15	16	17	18	20	22	24			
	Hb (g/dl)	11.7	11.4	10.9	11.4	11.2	11.3	11.5	11.6	10.8	11.3	10.6	10.9	10.7	10.8	10.8	10.1	10.1			
	PLT (x 10^3/μl)	257	322	330	343	427	457	525	493		502	405	385	332	328	278	237	217			
	WBC (/μl)	4700	5300	5200	6200	4600	4800	4000	3200	4300	4400	4100	4100	3600	4200	4000	3900	3600			
	neutrophil (/μl)	3304	3297	3682	4737	2594	2486	1692	966	2189	2270	2189	1193	1202	1256	1300	1299	1040			
	lymphocyte (/μl)	1161	1489	941	980	1490	1819	1844	1840	1733	1694	1427	2464	2027	2482	2248	2153	2149			
	monocyte (/μl)	207	445	499	415	432	365	372	346	292	326	373	332	270	332	308	269	234			
	neutrophils (%)	70.3	62.2	70.8	76.4	56.4	51.8	42.3	30.2	50.9	51.6	53.4	29.1	33.4	29.9	32.5	33.3	28.9			
	lymphocytes (%)	24.7	28.1	18.1	15.8	32.4	37.9	46.1	57.5	40.3	38.5	34.8	60.1	56.3	59.1	56.2	55.2	59.7			
	monocytes (%)	4.4	8.4	9.6	6.7	9.4	7.6	9.3	10.8	6.8	7.4	9.1	8.1	7.5	7.9	7.7	6.9	6.5			
P05	Days after symptom onset	5	6	7	8	9	10	11	12	13	14	15	16	17	18	20	22	24			
	Hb (g/dl)	12.6	11.7	11.6	11.6	12.6	12	12.2	11.8	11.5	11.6	12	11	11.6	10.6	10.7	10.8	9.8			
	PLT (x 10^3/μl)	175	204	228	249	289	323	371	395	401	425	458	389	330	331	400	416	368			
	WBC (/μl)	3400	4400	3700	4400	4800	4000	3800	4350	3900	3800	2800	3300	2600	3100	3400	3400	3100			
	neutrophil (/μl)	2615	3480	2738	3705	3917	3260	2820	2984	2968	2827	1722	2241	1342	880	1309	1649	1553			
	lymphocyte (/μl)	561	576	692	466	552	504	657	835	445	509	633	627	660	1646	1482	1221	1026			
	monocyte (/μl)	211	334	252	207	149	212	300	492	417	388	400	393	572	552	575	473	446			
	neutrophils (%)	76.9	79.1	74	84.2	81.6	81.5	74.2	68.6	76.1	74.4	61.5	67.9	51.6	28.4	38.5	48.5	50.1			
	lymphocytes (%)	16.5	13.1	18.7	10.6	11.5	12.6	17.3	19.2	11.4	13.4	22.6	19	25.4	53.1	43.6	35.9	33.1			
	monocytes (%)	6.2	7.6	6.8	4.7	3.1	5.3	7.9	11.3	10.7	10.2	14.3	11.9	22	17.8	16.9	13.9	14.4			
P06	Days after symptom onset	4	5	6	7	8	9	10	11	12	13	14	15	16	18	20	22	24	27		
	Hb (g/dl)	12.9	14	14	13.9	13.9	13.1	13.7	14.1	12.8	12.7	12.6	12.2	11.9	11.6	11	11	12.1	11.1		
	PLT (x 10^3/μl)	118	99	90	77	76	88	103	122	123	131	139	146	155	99	108	126	102	138		
	WBC (/μl)	2240	2300	1700	10000	8700	3000	1400	14200	10000	4800	2800	2300	3100	1900	1400	4800	2400	2800		
	neutrophil (/μl)	1496	1424	923	9050	8004	2328	720	13618	9190	3898	1814	1463	1721	1032	706	3950	1471	1145		
	lymphocyte (/μl)	511	688	559	620	513	468	494	525	630	566	566	481	694	496	374	379	410	1014		
	monocyte (/μl)	159	184	213	330	174	189	165	43	150	322	398	338	636	340	301	461	494	468		
	neutrophils (%)	66.8	61.9	54.3	90.5	92	77.6	51.4	95.9	91.9	81.2	64.8	63.6	55.5	54.3	50.4	82.3	61.3	40.9		
	lymphocytes (%)	22.8	29.9	32.9	6.2	5.9	15.6	35.3	3.7	6.3	11.8	20.2	20.9	22.4	26.1	26.7	7.9	17.1	36.2		
	monocytes (%)	7.1	8	12.5	3.3	2	6.3	11.8	0.3	1.5	6.7	14.2	14.7	20.5	17.9	21.5	9.6	20.6	16.7		
P07	Days after symptom onset	6	7	8	9	10	11	12	13	14	15	16	17	18	19	21	23	25	27	30	33
	Hb (g/dl)	12	11.3	11.4	11.4	11.9	12.6	12.8	11.3	12.8	13.6	10.9	10.1	9.5	8.1	10.2	10.4	11.5	10.9	11.4	11.2
	PLT (x 10^3/μl)	131	83	81	88	100	126	138	117	106	120	187	211	251	315	377	417	419	358	311	253
	WBC (/μl)	2000	1600	1600	1600	1700	1500	2400	1800	1900	1600	2700	2300	2080	1600	1900	2400	2300	2400	2500	2380
	neutrophil (/μl)	1208	779	846	981	1000	948	1843	1354	1549	1245	2138	1757	1387	707	874	1027	906	986	1233	1031
	lymphocyte (/μl)	642	691	638	533	564	489	434	335	207	240	397	382	437	626	661	876	1026	1061	903	990
	monocyte (/μl)	150	118	107	80	128	60	120	106	143	110	159	147	227	240	327	439	331	326	298	309
	neutrophils (%)	60.4	48.7	52.9	61.3	58.8	63.2	76.8	75.2	81.5	77.8	79.2	76.4	66.7	44.2	46	42.8	39.4	41.1	49.3	43.3
	lymphocytes (%)	32.1	43.2	39.9	33.3	33.2	32.6	18.1	18.6	10.9	15	14.7	16.6	21	39.1	34.8	36.5	44.6	44.2	36.1	41.6
	monocytes (%)	7.5	7.4	6.7	5	7.5	4	5	5.9	7.5	6.9	5.9	6.4	10.9	15	17.2	18.3	14.4	13.6	11.9	13
P08	Days after symptom onset	12	13	14	15	16	17	18	19	20	21	22	23	28							
	Hb (g/dl)	13.2	13.7	12.1	12.9	12.9	13	13.2	12.4	12.5	12.3	12.7	11.7	12.3							
	PLT (x 10^3/μl)	155	167	179	204	225	260	277	294	348	357	398	410	403							
	WBC (/μl)	1910	7700	9300	6200	5200	7400	9000	9100	8200	8900	7900	5000	5200							
	neutrophil (/μl)	1117	6691	8351	5134	4118	6098	7200	7398	6396	6880	5878	3270	3401							
	lymphocyte (/μl)	623	747	632	670	614	614	837	683	689	1015	1122	1160	1399							
	monocyte (/μl)	166	262	307	391	442	659	909	974	1099	970	869	540	281							
	neutrophils (%)	58.5	86.9	89.8	82.8	79.2	82.4	80	81.3	78	77.3	74.4	65.4	65.4							
	lymphocytes (%)	32.6	9.7	6.8	10.8	11.8	8.3	9.3	7.5	8.4	11.4	14.2	23.2	26.9							
	monocytes (%)	8.7	3.4	3.3	6.3	8.5	8.9	10.1	10.7	13.4	10.9	11	10.8	5.4							
P09	Days after symptom onset	7	8	9	10	11	12	13	14	15	16	17	18	19	20	22	24	26	28	31	34
	Hb (g/dl)	11.5	9.9	9.8	15.4	13.6	12.9	12.8	12.3	12	8.4	11.2	10.4	9.8	11.5	10.4	9.2	8.4	9.7	8.9	9.2
	PLT (x 10^3/μl)	165	157	177	85	81	62	68	70	73	134	121	146	114	133	198	190	195	203	157	129
	WBC (/μl)	4100	4600	4430	6400	7800	6100	7000	9000	15000	15700	14200	14200	11600	12600	13800	11200	10300	10600	7970	6700
	neutrophil (/μl)	3395	3689	3367	5126	6248	4721	5943	7218	12900	12968	11516	11658	10452	10710	10902	8994	7251	7961	5212	4536
	lymphocyte (/μl)	631	787	820	992	1014	604	448	621	600	1743	1420	1108	615	832	1076	1075	1494	1367	1347	1240
	monocyte (/μl)	57	110	235	243	460	634	518	1035	1440	926	1221	1363	487	1021	1794	918	1123	954	271	690
	neutrophils (%)	82.8	80.2	76	80.1	80.1	77.4	84.9	80.2	86	82.6	81.1	82.1	90.1	85	79	80.3	70.4	75.1	65.4	67.7
	lymphocytes (%)	15.4	17.1	18.5	15.5	13	9.9	6.4	6.9	4	11.1	10	7.8	5.3	6.6	7.8	9.6	14.5	12.9	16.9	18.5
	monocytes (%)	1.4	2.4	5.3	3.8	5.9	10.4	7.4	11.5	9.6	5.9	8.6	9.6	4.2	8.1	13	8.2	10.9	9	3.4	10.3
P10	Days after symptom onset	4	5	6	7	8	9	10	11	12	13										
	Hb (g/dl)	12.8	13.8	14.1	14.3	14.3	12.8	12.6	12.5	12.5	12.8										
	PLT (x 10^3/μl)	135	140	112	108	119	113	125	138	145	146										
	WBC (/μl)	3600	4200	4500	5100	7300	8200	6000	4000	4200	5600										
	neutrophil (/μl)	2365	2478	3497	4304	6424	7724	5436	3524	3583	4878										
	lymphocyte (/μl)	842	1365	698	530	650	303	348	244	281	381										
	monocyte (/μl)	364	336	288	245	226	172	210	224	328	342										
	neutrophils (%)	65.7	59	77.7	84.4	88	94.2	90.6	88.1	85.3	87.1										
	lymphocytes (%)	23.4	32.5	15.5	10.4	8.9	3.7	5.8	6.1	6.7	6.8										
	monocytes (%)	10.1	8	6.4	4.8	3.1	2.1	3.5	5.6	7.8	6.1										
P11	Days after symptom onset	5	6	7	8	9	10	11	12	13	14	15	16	17							
	Hb (g/dl)	12.1	11.7	12.6	12.4	14.2	11.7	12.8	12.4	12.6	11.9	10.9	11.2	11.3							
	PLT (x 10^3/μl)	233	183	166	141	144	124	132	127	118	136	98	88	74							
	WBC (/μl)	6740	5000	4600	4900	4500	3800	3900	4700	5800	6200	5200	5900	8500							
	neutrophil (/μl)	4671	3600	3137	2940	2439	2595	2668	3431	4472	5034	4316	4773	7293							
	lymphocyte (/μl)	1186	775	837	1284	1553	749	800	832	870	601	411	779	799							
	monocyte (/μl)	816	605	589	652	504	429	410	428	458	552	468	336	332							
	neutrophils (%)	69.3	72	68.2	60	54.2	68.3	68.4	73	77.1	81.2	83	80.9	85.8							
	lymphocytes (%)	17.6	15.5	18.2	26.2	34.5	19.7	20.5	17.7	15	9.7	7.9	13.2	9.4							
	monocytes (%)	12.1	12.1	12.8	13.3	11.2	11.3	10.5	9.1	7.9	8.9	9	5.7	3.9							
P12	Days after symptom onset	4	6	7	8	9	10	11	12	13	14	15	16	17							
	Hb (g/dl)	14.3	15.5	14	14.9	15.3	15.4	14.1	13.8	15.4	14.9	12.6	12.9	12.1							
	PLT (x 10^3/μl)	184	170	134	110	110	101	94	89	99	59	108	91	39							
	WBC (/μl)	7000	8500	7300	9500	8100	8300	5600	6500	10600	15600	18200	15700	9950							
	neutrophil (/μl)	3969	4726	5241	7334	5889	5968	3713	4674	7558	14399	16817	14130	8627							
	lymphocyte (/μl)	1911	2635	1409	1710	1337	1569	1338	1118	912	499	455	612	547							
	monocyte (/μl)	868	969	584	380	745	755	510	683	1049	686	910	895	766							
	neutrophils (%)	56.7	55.6	71.8	77.2	72.7	71.9	66.3	71.9	71.3	92.3	92.4	90	86.7							
	lymphocytes (%)	27.3	31	19.3	18	16.5	18.9	23.9	17.2	8.6	3.2	2.5	3.9	5.5							
	monocytes (%)	12.4	11.4	8	4	9.2	9.1	9.1	10.5	9.9	4.4	5	5.7	7.7							
P13	Days after symptom onset	3	4	5	6	7	8	9	10	11	12	13									
	Hb (g/dl)	12	12	12	12.8	12.7	12.2	13.4	12.9	13.6	13.2	12.6									
	PLT (x 10^3/μl)	189	168	167	165	159	171	232	201	209	160	74									
	WBC (/μl)	5500	8600	8200	6000	6100	3800	8600	6100	10500	12200	15800									
	neutrophil (/μl)	5236	7405	7019	4698	5728	2960	6923	5435	9345	10809	15089									
	lymphocyte (/μl)	198	688	771	996	165	312	327	165	210	232	174									
	monocyte (/μl)	44	490	410	276	201	505	1342	500	945	1159	537									
	neutrophils (%)	95.2	86.1	85.6	78.3	93.9	77.9	80.5	89.1	89	88.6	95.5									
	lymphocytes (%)	3.6	8	9.4	16.6	2.7	8.2	3.8	2.7	2	1.9	1.1									
	monocytes (%)	0.8	5.7	5	4.6	3.3	13.3	15.6	8.2	9	9.5	3.4									
P14	Days after symptom onset	3	4	5	6	7	8	9	10	11	12										
	Hb (g/dl)	12	11	11.2	11.6	11.3	11.9	11.6	10	10	11.5										
	PLT (x 10^3/μl)	111	77	69	67	60	72	73	51	45	94										
	WBC (/μl)	8400	5000	3300	2400	3200	5900	8600	7400	9390	9800										
	neutrophil (/μl)	8072	4560	2802	1879	3011	5505	8024	7089	9108	9633										
	lymphocyte (/μl)	277	365	383	422	170	307	387	207	207	59										
	monocyte (/μl)	42	60	112	91	19	71	181	104	75	98										
	neutrophils (%)	96.1	91.2	84.9	78.3	94.1	93.3	93.3	95.8	97	98.3										
	lymphocytes (%)	3.3	7.3	11.6	17.6	5.3	5.2	4.5	2.8	2.2	0.6										
	monocytes (%)	0.5	1.2	3.4	3.8	0.6	1.2	2.1	1.4	0.8	1										

Supplementary Table S4. Kinetic responses of 39 cytokines, chemokines, and growth factors in MERS patients.

		All in pg/ml ext RANTES (ng/ml)	Type 1 IFN	Hematopoiesis				Inflammatory cytokines						
Groups	ID	Days after symptom onset	IFN-α2	Flt-3L	G-CSF	GM-CSF	IL-3	IL-1RA	IL-1α	IL-1β	TNF-α	IL-6	IL-10	TGF-β
Revered	P01	2	61.9	3.2	26.4	0.0	0.0	40.4	0.0	0.0	27.7	3.8	3.7	779.3
		9	100.2	0.0	22.3	9.9	0.0	14.0	4.3	7.0	25.9	0.0	1.1	105.8
	P02	3	142.3	0.0	72.2	29.5	0.0	10.2	115.2	2.9	19.5	11.4	4.4	25.4
		8	18.5	0.0	3.9	0.0	0.0	19.6	0.0	0.0	20.2	0.0	0.0	105.1
		15	90.8	0.0	26.4	11.6	0.0	11.3	40.2	4.6	22.1	2.0	0.0	487.6
	P03	4	212.9	0.0	52.9	13.6	0.0	9.2	140.2	2.2	19.7	11.9	8.4	541.0
		10	73.9	17.1	30.0	8.2	0.0	3.2	80.1	2.5	20.1	26.6	3.3	52.6
		17	28.4	0.0	123.6	183.1	0.0	8.4	0.0	371.0	148.9	2315.0	0.0	89.8
	P04	5	48.9	7.4	13.6	16.3	0.0	0.0	257.6	5.3	18.3	39.2	7.7	0.0
		8	248.7	2.6	3.9	2.9	0.0	72.5	35.5	0.0	24.6	21.0	16.0	24.1
		15	333.6	5.5	4.8	7.5	0.0	0.0	80.1	3.9	32.8	69.3	1.5	29.6
	P05	5	218.2	11.6	41.0	0.0	0.0	42.0	15.9	0.0	25.4	40.6	46.5	108.3
		8	522.2	0.0	28.2	0.0	0.0	35.1	0.0	0.0	24.5	38.2	11.5	21.5
		13	348.7	0.0	2.7	0.0	0.0	3.7	0.0	0.0	34.7	9.1	7.9	79.4
		20	280.7	0.0	8.9	0.0	0.0	3.7	0.0	0.0	31.4	4.2	3.9	80.0
		25	115.5	0.0	15.1	0.0	0.0	4.7	63.6	0.0	32.3	0.0	21.7	152.4
	P06	9	354.3	0.0	102.2	4.5	0.0	23.1	17.1	0.0	43.1	46.6	12.1	736.9
		17	187.8	0.0	41.9	7.5	0.0	6.5	0.0	0.0	32.2	83.9	11.3	41.8
		26	318.8	0.0	0.0	0.0	0.0	20.0	0.0	0.0	43.5	20.5	0.0	42.0
		34	139.9	0.0	7.6	0.0	0.0	30.5	0.0	10.7	69.4	74.2	3.2	34.9
	P07	11	320.1	0.0	33.6	0.0	0.0	3.2	0.0	0.0	43.5	33.2	22.2	9.2
		14	198.1	0.0	38.0	0.0	0.0	33.6	0.0	0.0	35.5	50.0	50.7	1931.3
		21	60.9	0.0	8.2	9.0	0.0	8.0	0.0	0.0	26.3	4.1	6.5	92.9
		28	29.8	0.0	36.2	42.0	0.0	10.2	0.0	485.4	230.9	1323.0	3.3	70.4
		34	72.6	0.0	0.0	18.3	0.0	2.0	0.0	0.0	27.0	7.1	10.6	0.0
	P08	18	0.0	0.0	0.0	0.0	0.0	79.1	0.0	0.0	59.6	0.0	8.9	9258.1
		26	35.7	0.0	15.5	9.9	0.0	26.4	8.3	0.0	25.9	0.0	1.5	2754.1
		34	0.0	8.0	2.3	6.4	0.0	6.8	0.0	16.5	34.9	0.0	0.0	255.9
	P09	7	112.0	0.0	124.7	10.1	3.8	124.9	78.9	0.0	47.0	298.6	80.3	29.9
		10	168.7	0.0	49.2	4.0	0.0	137.7	10.5	0.0	66.1	222.2	47.6	29.8
		17	18.5	0.0	0.0	26.5	0.0	0.0	0.0	0.0	13.7	3.9	109.7	140.1
		24	98.7	0.0	15.5	45.3	0.0	12.6	0.0	0.0	24.1	23.1	36.9	19.8
Deceased	P10	4	362.7	0.0	68.7	16.9	0.0	47.9	141.3	2.0	26.6	11.9	18.7	86.4
		12	200.9	0.0	110.1	0.0	0.0	16.4	22.7	0.0	46.1	58.2	30.6	21.7
	P11	13	243.5	0.0	58.7	24.7	0.0	7.6	225.0	5.2	7.3	103.4	27.0	15.6
		17	139.2	0.0	190.3	25.9	0.0	3.9	178.8	3.4	5.4	218.9	29.1	13.8
	P12	6	31.0	0.0	40.2	0.0	0.0	37.2	33.2	0.0	17.8	37.1	8.9	3.2
		14	281.7	0.0	0.0	0.0	0.0	188.3	46.1	0.0	11.4	99.6	236.4	25.7
	P13	8	145.3	0.0	20.9	0.0	0.0	51.1	21.6	0.0	27.1	10.6	176.0	19.2
		13	0.0	0.0	1086.0	0.0	0.0	310.0	0.0	0.0	169.8	590.6	247.4	3.2
	P14	8	425.1	0.0	20.5	0.0	0.0	0.0	7.3	0.0	18.0	6.3	17.4	0.0
		13	205.6	0.0	159.5	0.0	0.0	4.2	0.0	0.0	28.9	88.4	24.7	7.4

			Chemokines										
Groups	ID	Days after symptom onset	Fractalkine	MCP-1	MCP-3	IP-10	MDC	RANTES	IL-8	MIP-1α	MIP-1β	Eotaxin	GRO
Revered	P01	2	44.2	843.4	0.0	2373.0	242.7	33.2	34.7	0.0	71.3	304.6	6541.0
		9	125.3	518.8	0.0	464.4	247.0	42.4	145.7	35.2	74.6	290.0	9032.0
	P02	3	358.5	521.4	0.0	1505.0	323.1	29.1	7.4	18.2	60.8	223.5	1479.0
		8	0.0	388.0	0.0	767.6	273.2	43.4	12.1	9.7	60.0	244.7	15136.0
		15	259.7	469.9	0.0	504.1	340.3	32.8	170.8	144.3	118.6	321.0	3091.0
	P03	4	254.3	531.9	0.0	12422.0	460.6	25.4	8.1	7.1	40.0	204.9	12659.0
		10	192.6	350.2	0.0	945.7	465.4	30.0	56.8	18.8	44.4	172.4	2077.0
		17	0.0	5818.0	1003.0	972.6	440.6	32.4	5764.0	643.3	250.1	299.1	15136.0
	P04	5	186.7	278.2	0.0	11528.0	561.0	9.0	10.9	0.0	43.6	63.2	529.3
		8	0.0	304.9	0.0	16706.0	422.1	17.2	15.5	0.0	28.4	52.9	1108.0
		15	82.5	1917.0	122.1	4289.0	848.3	29.5	1077.0	118.5	175.7	199.3	15136.0
	P05	5	0.0	547.5	0.0	19498.0	505.7	21.5	15.3	5.8	19.8	96.7	1256.0
		8	0.0	734.9	0.0	21365.0	340.8	19.3	28.5	0.0	19.6	115.8	794.8
		13	0.0	737.8	0.0	2832.0	218.5	40.9	41.3	12.3	42.1	136.3	15136.0
		20	88.8	996.5	195.5	6771.0	381.0	34.9	919.5	16.5	138.6	372.3	15136.0
		25	240.6	906.3	0.0	17494.0	818.9	32.0	160.0	21.8	97.2	329.9	5273.0
	P06	9	37.0	1489.0	0.0	19006.0	438.2	17.7	51.4	28.2	49.9	218.1	877.0
		17	82.5	380.3	0.0	3486.0	405.4	42.8	40.2	5.8	31.7	80.3	2472.0
		26	0.0	1774.0	250.9	3048.0	321.3	20.8	585.6	94.0	185.1	317.7	4241.0
		34	65.9	2148.0	157.6	1938.0	574.3	33.3	3537.0	280.1	366.0	316.6	15136.0
	P07	11	0.0	1127.0	0.0	17745.0	518.9	12.5	23.8	0.0	21.9	87.8	973.5
		14	0.0	1139.0	0.0	17829.0	381.0	10.7	18.5	0.0	0.0	91.8	602.2
		21	44.2	627.3	0.0	1371.0	375.6	33.2	33.2	0.0	0.0	120.9	1192.0
		28	72.2	2034.0	1056.0	1928.0	468.8	25.4	4872.0	1931.0	135.4	309.7	2485.0
		34	0.0	777.2	49.5	1754.0	338.8	3.3	255.0	57.0	39.4	295.5	999.4
	P08	18	0.0	250.2	0.0	3567.0	667.0	24.5	25.6	14.4	48.5	49.7	1899.0
		26	204.2	220.8	0.0	1709.0	660.8	42.2	12.2	8.6	49.8	87.3	15136.0
		34	301.6	1997.0	458.2	1499.0	376.6	31.4	4098.0	311.1	231.3	224.0	15136.0
	P09	7	58.3	9446.0	685.7	27260.0	382.9	17.5	181.0	49.9	58.5	487.7	15136.0
		10	1256.0	7811.0	573.6	23685.0	270.9	18.4	254.7	48.8	62.0	853.1	15136.0
		17	0.0	546.8	648.2	729.5	111.3	21.2	2416.0	43.3	35.0	234.0	4922.0
		24	0.0	669.5	0.0	5280.0	85.8	5.9	102.9	9.3	22.2	468.4	3227.0
Deceased	P10	4	423.4	814.3	0.0	18868.0	472.7	16.7	16.7	13.8	26.3	232.5	1095.0
		12	254.3	1156.0	76.9	22353.0	378.9	27.5	35.1	27.0	0.0	175.3	15136.0
	P11	13	51.3	913.8	0.0	10856.0	598.7	8.2	234.5	8.6	12.5	269.8	743.0
		17	79.1	1841.0	0.0	16148.0	411.0	20.6	249.6	10.3	8.2	364.3	1568.0
	P12	6	0.0	437.4	0.0	4457.0	681.9	13.9	37.7	0.0	10.3	167.2	383.0
		14	0.0	721.2	0.0	6424.0	313.0	19.8	165.0	0.0	12.3	216.0	1048.0
	P13	8	134.9	699.0	0.0	22426.0	307.2	13.5	48.7	0.0	0.0	138.6	797.8
		13	1574.0	1768.0	0.0	15149.0	174.8	18.1	285.6	42.1	159.0	108.5	1689.0
	P14	8	0.0	733.6	0.0	18826.0	152.9	3.6	45.1	0.0	0.0	144.9	573.3
		13	0.0	1851.0	0.0	8674.0	117.4	11.2	83.7	13.3	0.0	106.0	942.8

			T cell responses										Platelet	Growth factors			
Groups	ID	Days after symptom onset	IL-2	IL-7	IL-15	IL-12p70	IL-12p40	IFN-γ	IL-4	IL-5	IL-9	IL-17A	sCD40L	TGF-α	EGF	FGF-2	VEGF
Revered	P01	2	0.0	3.8	0.9	0.0	0.0	29.2	0.0	0.0	0.0	0.0	3742.0	0.7	119.3	63.4	0.0
		9	0.0	7.1	0.0	1.1	0.0	2.7	0.0	0.0	0.0	0.0	4636.0	6.9	267.2	120.7	249.4
	P02	3	0.0	9.7	3.9	11.9	30.5	11.5	28.9	1.0	1.6	3.9	1090.0	2.9	77.3	148.5	509.7
		8	0.0	3.5	0.0	0.9	0.0	4.3	0.0	0.0	0.0	2.8	7382.0	0.9	341.8	31.9	368.7
		15	0.0	4.8	0.8	6.3	0.0	6.3	10.9	0.0	0.0	2.8	3792.0	4.2	344.5	111.3	467.5
	P03	4	0.0	13.1	4.9	4.3	6.4	21.6	0.0	0.0	0.0	1.7	2412.0	1.7	90.5	131.8	172.2
		10	0.0	10.5	1.8	2.4	0.0	6.6	0.0	0.0	0.0	1.2	4453.0	2.7	99.7	114.2	127.0
		17	0.0	11.3	1.1	0.1	0.0	1.1	0.0	0.0	0.0	0.0	2291.0	0.8	148.2	17.4	79.7
	P04	5	8.6	0.0	5.5	3.0	0.0	25.2	0.0	0.0	0.0	8.4	326.8	3.5	89.7	45.0	0.0
		8	0.0	0.0	9.2	0.0	0.0	55.3	0.0	1.5	0.0	0.0	1910.0	8.1	100.4	0.0	0.0
		15	1.1	0.0	2.0	1.1	0.0	12.0	0.0	0.0	0.0	5.1	5531.0	6.0	329.5	27.2	0.0
	P05	5	0.0	0.0	9.2	0.1	0.0	30.5	0.0	0.0	0.0	0.0	1117.0	1.8	45.3	0.0	0.0
		8	0.0	0.0	11.1	0.0	0.0	10.1	0.0	0.0	0.0	0.0	1675.0	5.8	47.5	0.0	0.0
		13	0.0	8.3	5.2	0.0	0.0	0.0	0.0	0.0	1.0	0.0	6222.0	3.2	258.6	99.1	232.9
		20	0.0	9.7	3.7	0.5	0.0	1.0	0.0	0.0	0.0	0.0	4500.0	0.5	299.8	0.0	145.1
		25	0.0	6.5	3.3	1.1	0.0	4.9	3.4	1.2	0.0	0.0	3814.0	0.7	267.2	24.8	79.7
	P06	9	0.0	0.0	14.2	0.1	0.0	8.4	0.0	0.0	1.1	16.1	1754.0	0.2	26.9	45.0	399.7
		17	0.0	5.2	6.0	0.9	0.0	3.5	0.0	3.4	0.0	15.6	5588.0	1.0	206.4	137.7	818.9
		26	0.0	0.0	0.0	0.0	0.0	3.0	0.0	14.9	0.0	15.4	1629.0	1.5	77.1	26.0	237.1
		34	0.0	6.5	1.6	0.2	0.0	4.0	0.0	0.0	0.0	23.4	7588.0	1.0	245.7	47.7	538.8
	P07	11	0.0	0.0	6.1	0.1	0.0	9.3	0.0	0.0	0.0	4.6	800.9	0.2	52.9	19.1	0.0
		14	0.0	0.0	6.4	0.0	0.0	14.6	0.0	0.0	0.0	2.8	1334.0	2.7	44.2	0.0	0.0
		21	0.0	4.2	1.1	0.9	0.0	1.5	0.0	1.0	0.0	0.0	2540.0	0.3	276.3	148.5	253.5
		28	0.0	11.0	0.0	0.2	0.0	2.5	0.0	0.0	0.0	3.6	1843.0	1.4	181.7	12.6	192.2
		34	0.0	0.0	1.6	1.1	0.0	2.4	0.0	0.0	0.0	3.3	263.3	17.8	9.6	0.0	0.0
	P08	18	0.0	0.0	3.9	0.0	0.0	3.9	0.0	2.3	0.0	0.0	2781.0	22.0	79.9	43.2	0.0
		26	0.0	5.0	1.5	0.5	0.0	3.3	0.0	0.0	0.0	0.0	5406.0	3.3	233.6	145.3	215.5
		34	0.0	4.6	0.8	0.0	0.0	3.3	0.0	0.0	0.0	1.4	7196.0	12.0	254.9	62.0	86.0
	P09	7	0.0	0.0	8.6	0.1	11.6	337.7	0.0	1.5	2.5	2.2	4226.0	29.4	392.4	20.6	920.9
		10	0.0	0.0	10.6	0.0	0.0	164.0	0.0	0.0	1.7	0.0	1396.0	17.4	142.0	0.0	696.4
		17	0.0	0.0	7.5	0.0	0.0	0.0	0.0	0.0	0.0	1.5	1211.0	48.8	99.6	0.0	0.0
		24	0.0	0.0	11.0	0.0	0.0	1.1	0.0	0.0	0.0	0.0	440.8	36.0	54.2	0.0	0.0
Deceased	P10	4	0.0	8.7	16.0	3.6	6.7	28.0	19.0	0.0	1.0	2.6	668.5	4.7	44.4	106.9	331.9
		12	0.0	9.4	17.3	0.0	0.0	7.9	0.0	0.0	1.2	0.0	1968.0	3.9	102.7	122.2	651.5
	P11	13	4.2	3.3	17.1	0.0	14.2	16.0	0.0	0.0	0.0	4.9	422.9	9.5	54.3	56.0	127.0
		17	3.1	3.0	21.0	0.0	0.0	11.4	0.0	0.0	0.0	1.4	1416.0	19.4	86.9	68.9	0.0
	P12	6	0.0	0.0	0.0	0.0	0.0	1.3	0.0	0.0	0.0	0.0	932.5	7.0	24.7	0.0	0.0
		14	0.0	0.0	0.6	0.0	0.0	3.3	0.0	0.0	0.0	0.0	1414.0	4.2	57.7	31.9	0.0
	P13	8	0.0	8.0	8.4	0.0	0.0	12.3	0.0	0.0	0.0	0.0	2045.0	3.8	91.3	0.0	1022.0
		13	0.0	0.0	24.4	0.0	0.0	0.0	0.0	0.0	0.0	0.0	728.0	10.0	29.0	0.0	0.0
	P14	8	0.0	0.0	6.0	0.0	0.0	4.9	0.0	0.0	0.0	1.5	167.8	9.2	0.0	0.0	0.0
		13	0.0	0.0	11.1	0.3	0.0	5.4	0.0	0.0	0.0	1.7	274.1	14.7	0.0	0.0	0.0

Supplementary Table S5. Hazards ratios and 95% confidence intervals of mortality according to the virological and immunological factors. If HR > 1.1 in red, <0.9 in blue.

model	A. crude, since exposed				B. age-adjusted, since exposed			
variable	HR	95% CI		p value	HR	95% CI		p value
resp_virus	1.0890	0.7969	1.4881	0.5930	0.9767	0.7372	1.2938	0.8690
plasma_virus	2.9895	0.4774	18.7219	0.2420	5.7860	0.1681	199.1157	0.3310
Serum IgG	0.6423	0.3919	1.0527	0.0790	0.9202	0.5127	1.6518	0.7810
Respiratory IgA (nOD)	0.0816	0.0066	1.0024	0.0500	0.0328	0.0007	1.5839	0.0840
Respiratory IgA (titer)	0.9884	0.9755	1.0015	0.0840	0.9941	0.9791	1.0095	0.4510
leukocytes counts	1.0005	1.0001	1.0008	0.0110	1.0005	1.0001	1.0009	0.0260
neutrophil counts	1.0005	1.0001	1.0009	0.0140	1.0005	1.0001	1.0010	0.0280
lymphocyte counts	0.9954	0.9909	0.9999	0.0440	0.9969	0.9927	1.0011	0.1490
monocyte counts	1.0011	0.9971	1.0051	0.5910	1.0025	0.9976	1.0074	0.3220
platelete counts	0.9634	0.9279	1.0003	0.0520	0.9166	0.8179	1.0273	0.1340
ifna2	0.9999	0.9928	1.0070	0.9770	0.9992	0.9897	1.0088	0.8710
il1ra	1.0097	0.9997	1.0198	0.0570	1.0082	0.9985	1.0181	0.0990
il1a	1.0035	0.9906	1.0166	0.6000	1.0174	0.9961	1.0391	0.1110
il1b	0.8884	0.4967	1.5892	0.6900	0.9865	0.6976	1.3952	0.9390
tnfa	1.0061	0.9879	1.0246	0.5170	1.0082	0.9875	1.0294	0.4380
il6	1.0000	0.9984	1.0017	0.9720	1.0013	0.9991	1.0035	0.2490
il10	1.0126	1.0017	1.0236	0.0240	1.0106	0.9995	1.0219	0.0610
tgfb	0.8763	0.7695	0.9978	0.0460	0.8431	0.6945	1.0234	0.0840
fractalkine	1.0014	0.9997	1.0030	0.1110	1.0010	0.9991	1.0028	0.2980
mcp1	0.9999	0.9995	1.0004	0.8210	1.0001	0.9995	1.0006	0.8340
mcp3	0.9953	0.9814	1.0095	0.5150	0.9777	0.9352	1.0222	0.3210
ip10	1.0001	1.0000	1.0002	0.2260	1.0000	0.9999	1.0002	0.6460
mdc	0.9936	0.9862	1.0011	0.0960	0.9957	0.9873	1.0042	0.3170
rantes	0.8998	0.7977	1.0149	0.0860	0.8639	0.7451	1.0017	0.0530
il8	0.9994	0.9972	1.0017	0.6080	0.9998	0.9975	1.0021	0.8680
mip1a	0.9928	0.9645	1.0219	0.6220	0.9846	0.9380	1.0335	0.5300
mip1b	0.9906	0.9692	1.0124	0.3940	0.9986	0.9783	1.0193	0.8930
eotaxin	0.9987	0.9915	1.0059	0.7200	0.9993	0.9902	1.0084	0.8730
gro	0.9999	0.9997	1.0001	0.3250	0.9998	0.9995	1.0000	0.0910
il2	1.2349	0.6295	2.4227	0.5390	2.9259	0.9706	8.8201	0.0570
il7	0.9158	0.7004	1.1976	0.5210	0.7405	0.5081	1.0792	0.1180
il15	1.1464	1.0216	1.2865	0.0200	1.1312	0.9906	1.2917	0.0690
il12p70	0.0544	0.0002	14.6187	0.3080	0.0445	0.0002	8.0722	0.2410
ifng	0.9339	0.7912	1.1023	0.4190	0.9284	0.7560	1.1401	0.4780
il9	0.7857	0.1651	3.7388	0.7620	0.2067	0.0139	3.0788	0.2530
il17a	0.7218	0.3603	1.4460	0.3580	0.7958	0.4080	1.5523	0.5030
scd40l	0.9981	0.9960	1.0002	0.0830	0.9981	0.9959	1.0003	0.0940
gcsf	1.0029	1.0002	1.0057	0.0370	1.0024	0.9995	1.0052	0.1040
gmcsf	0.9762	0.8838	1.0781	0.6340	1.0032	0.9465	1.0632	0.9150
tgfa	1.0201	0.9468	1.0990	0.6010	1.0368	0.9605	1.1191	0.3540
egf	0.9633	0.9303	0.9974	0.0350	0.9652	0.9303	1.0014	0.0590
fgf2	0.9945	0.9751	1.0142	0.5810	0.9843	0.9627	1.0065	0.1640
vegf	0.9986	0.9947	1.0025	0.4820	0.9949	0.9890	1.0008	0.0880

p-value <0.05

p-value <0.10
